# Accurate regional influenza epidemics tracking using Internet search data

**DOI:** 10.1038/s41598-019-41559-6

**Published:** 2019-03-27

**Authors:** Shaoyang Ning, Shihao Yang, S. C. Kou

**Affiliations:** Department of Statistics, Harvard University, 1 Oxford Street, Cambridge, 02138 MA USA

## Abstract

Accurate, high-resolution tracking of influenza epidemics at the regional level helps public health agencies make informed and proactive decisions, especially in the face of outbreaks. Internet users’ online searches offer great potential for the regional tracking of influenza. However, due to the complex data structure and reduced quality of Internet data at the regional level, few established methods provide satisfactory performance. In this article, we propose a novel method named ARGO2 (2-step Augmented Regression with GOogle data) that efficiently combines publicly available Google search data at different resolutions (national and regional) with traditional influenza surveillance data from the Centers for Disease Control and Prevention (CDC) for accurate, real-time regional tracking of influenza. ARGO2 gives very competitive performance across all US regions compared with available Internet-data-based regional influenza tracking methods, and it has achieved 30% error reduction over the best alternative method that we numerically tested for the period of March 2009 to March 2018. ARGO2 is reliable and robust, with the flexibility to incorporate additional information from other sources and resolutions, making it a powerful tool for regional influenza tracking, and potentially for tracking other social, economic, or public health events at the regional or local level.

## Introduction

**Figure 1:**
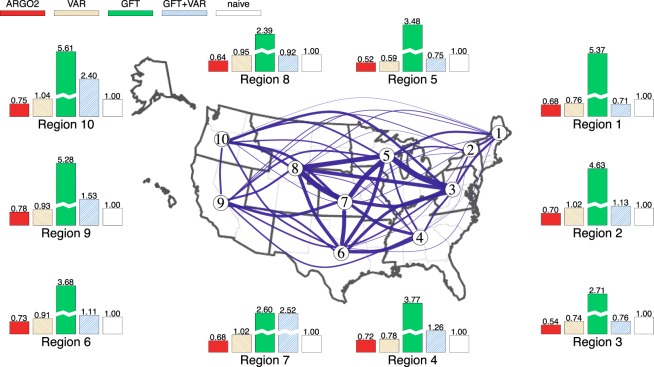
The ten US HHS regions: cross-regional correlation and estimation error breakdown. The US HHS regions include: Region 1 (CT, MA, ME, NH, RI, VT), Region 2 (NJ, NY), Region 3 (DE, MD, PA, VA, WV), Region 4 (AL, FL, GA, KY, MS, NC, SC, TN), Region 5 (IL, IN, MI, MN, OH, WI), Region 6 (AR, LA, NM, OK, TX), Region 7 (IA, KS, MO, NE), Region 8 (CO, MT, ND, SD, UT, WY), Region 9 (AZ, CA, HI, NV), and Region 10 (AK, ID, OR, WA). The width of the link between two regions corresponds to the strength of the correlation between two regions’ historic %ILI (wider represents higher correlation). The bars compare the region-by-region estimation error of ARGO2, VAR, GFT, GFT+VAR, and the naive method in terms of the relative MSE to the naive method for the period of March 29, 2009 to August 15, 2015. All methods and metrics in comparison are defined in Methods section.

Internet users’ online records contain the footprints of the activities of millions of individuals in nearly every aspect of life, and offer the potential for real-time tracking of public health and social events^1–3^, including influenza epidemics^4,5^, at the regional level^6–8^. Accurate, up-to-date regional tracking of influenza epidemics, which cause up to 500,000 deaths a year world-wide^9^, helps personnel including clinicians, epidemiologists, and public health officials, as well as relevant agencies to make informed and proactive decisions^10–12^, especially in face of outbreaks^13^. However, owing to the complexity of the data structure and low Internet data quality at the regional level, few existing methods provide satisfactory performance in estimating the regional flu activities^6,7,10,14–16^, which is of particular concern as most public health decisions, interventions, and resource allocations are conducted at the regional or local level. Here we present a novel method ARGO2 (standing for 2-step Augmented Regression with GOogle data) that gives accurate, real-time influenza tracking at the US Health and Human Services (HHS) regional level (see Fig. 1 for an illustration of the ten US HHS regions; e.g. Region 1 contains six northeastern states of US: CT, MA, ME, NH, RI, VT).

Traditionally, the situational awareness of influenza (flu) activities relies on the Influenza-like Illness (ILI) reports from nation-wide surveillance networks of healthcare centers. CDC’s Influenza-like Illness Surveillance Network (ILINet), in particular, aggregates the percentage of outpatients with ILI, and reports this ILI percentage (%ILI) at both the US national and the Health and Human Services (HHS) regional levels. Owing to the time needed for administrative aggregation and processing, CDC’s ILI report often lags behind real-time for 1 to 2 weeks (with less accuracy for more recent weeks), which is far from optimal for decision-making. To alleviate the lag of CDC’s ILI report, a new approach, digital disease detection, has been developed to provide decision makers with more systematic, objective, and timely information. Digital flu detection utilizes statistical or mechanistic models to estimate and forecast current and future %ILI at national and/or regional levels based on information from Internet-derived data, such as Google search data, as well as traditional surveillance data, such as CDC’s ILI reports. Examples of digital flu detection methods include Google Flu Trends (GFT)^5^, a susceptible-infectious-recovered-susceptible model with ensemble adjustment Kalman filter (SIRS-EAKF)^6,17–20^, an empirical Bayes framework with GFT^14^, an epidemiology model with GFT^8^, a wisdom-of-crowds human-based forecast^10^, an ensemble penalized regression^21^, an artificial-tree-enhanced neural network^22^, and a Gaussian process nonlinear estimator^23,24^, among others. Yang *et al*.^25,26^ proposed a method named ARGO (AutoRegression with GOogle search data) that works well at tracking %ILI at the US national level.

Despite the progress in digital flu detection over the last few years^27,28^, the regional %ILI estimation results, compared to the national ones, are considerably less accurate and still far from satisfactory, as documented in Biggerstaff *et al*.^11^. Furthermore, most public health decisions, interventions, and resource allocations (e.g., vaccine campaigns and outbreak responses) are carried out at the regional or local levels rather than at the national level. Many digital flu estimation methods^14,15,18,22^ apply the same method for both the national level estimation and the regional level estimation, even though the latter exhibits a strong spatial structure. Attempts have been made to consider the dependent geographical structure^12,28,29^; Zou *et al*.^29^, in particular, proposed a multi-task nonlinear regression method for regional estimation, which showed some good results. Nevertheless, most methods are still insufficient for accurate estimation of the %ILI at the US HHS regional level. In fact, many of the methods underperform a “naive” persistence forecast method. The naive method simply uses CDC’s reported regional %ILI of the previous week as the %ILI estimate for the current week without any modeling effort. See Supplementary Table S13 for further details on performance of methods in the 2015–2016 CDC’s Epidemic Prediction Initiative (FluSight challenge, predict.phiresearchlab.org, predict.cdc.gov).

To effectively address these difficulties, we introduce a novel method ARGO2 that gives accurate and robust real-time %ILI estimates at the regional level. ARGO2 introduces a statistically principled framework that combines multi-resolution, multi-source information with the regional dependence structure. It operates in two steps. In step one, ARGO2 extracts online search query information (using publicly available data from Google Trends) at two resolutions: at each individual region and at the national level. This information is then used to generate preliminary estimates for each region and for the nation as a whole. In step two, ARGO2 utilizes three components to boost performance: (1) the dependence structure between the different regions, (2) the temporal trend, and (3) the national estimate as the baseline. The two-step procedure of ARGO2 has the following features: (i) It automatically selects the most relevant search terms and filters out high-sparsity terms, which overcomes the lower-quality issue found in Google’s regional search data. It incorporates (ii) the lower-resolution, national %ILI estimate as the baseline, (iii) the short-term momentum of flu activity, and (iv) cross-regional influence (correlations) to boost estimation accuracy on high-resolution, regional estimation. (v) It adopts a two-year sliding window for model training, which intends to capture the evolution in people’s search patterns, Google’s search engine, epidemic activity, and other patterns that change over time^30^.

## Results

We apply ARGO2 to produce retrospective estimates of flu activity in the ten US HHS regions for the time period of March 29, 2009 to March 17, 2018. Our estimation target is CDC’s weekly weighted %ILI. In a given week, the most recent CDC’s ILI report typically reflects the %ILI of the previous week. At every week, to estimate the %ILI of the current week, we use the information that would have been available at that time, including the historical CDC’s ILI reports (which give the %ILI up to the previous week) and the flu-related search query volumes from Google Trends (which are available in real-time). We compare our estimates with the actual %ILI subsequently revealed by CDC weeks later and evaluate the estimation accuracy using multiple metrics, including mean squared error (MSE), mean absolute error (MAE), mean absolute percentage error (MAPE), and correlation with the true %ILI. We also compare the performance of our method with several benchmark methods (detailed in the Methods section), including: (i) GFT (last estimate available: the week ending on August 15, 2015), (ii) estimates by the lag-1 vector autoregressive model (VAR model), (iii) estimates by the lag-1 vector autoregressive model with regional GFT estimates as an exogenous variable, denoted as GFT + VAR, and (iv) naive estimates, which for each US HHS region simply use CDC’s reported %ILI of the previous week as the estimate for the current week. Since ARGO2 uses a two-year sliding window in its model training, for fair comparison, models (ii) and (iii) are also trained with the two-year sliding window. It is worth emphasizing that the methodology of ARGO2 was frozen on December 26, 2016, so 2017–2018 is a strict validation period for ARGO2.

We summarize the overall results in Fig. 1 and Table 1, averaging over ten US HHS regions. For MSE and MAE, ARGO2 uniformly outperforms all other methods for all flu seasons (2009–2018) considered in this study. ARGO2 also gives competitive performance in MAPEs and correlations. Specifically regarding MSE, ARGO2 statistically significantly outperforms all the alternatives at the 0.05 significance level (detailed in Supplementary Information and Supplementary Table S14): it gives over 35% error reduction from the naive method in MSE (ARGO2’s MSE = 0.149, 64.4% relative to naive, see also Supplementary Table S16), and 30% reduction from the best alternative method in comparison, i.e., VAR, (VAR’s MSE = 0.212) in the whole period of 2009–2018. Regarding MAE, ARGO2 reaches a 16% error reduction compared to the naive method (ARGO2’s MAE = 0.224, 83.6% relative to the naive method) in the whole period, and achieves a 12% improvement from the best alternative method in comparison (VAR’s MAE = 0.256). ARGO2 has the smallest MAPE in all the evaluation periods except the 2010–2011 flu season, and it holds the lead in the correlation metric in all evaluation periods except the 2009 H1N1 outbreak. Close examination of the H1N1 period shows that GFT significantly overshoots the true peak, resulting in spurious high correlation but poor error metrics, whereas ARGO2 performs consistently both in correlation and in the error metrics (Supplementary Figs S4–S13).

**Table 1:** Comparison of different methods for regional %ILI estimation.

	Whole period	‘09-’15	H1N1	‘10-’11	‘11-’12	‘12-’13	‘13-’14	‘14-’15	‘15-’16	‘16-’17	‘17-’18
**MSE**											
ARGO2	**0.149**	**0.165**	**0.628**	**0.121**	**0.047**	**0.240**	**0.101**	**0.248**	**0.059**	**0.157**	**0.278**
VAR	0.212	0.215	0.832	0.148	0.066	0.285	0.164	0.334	0.109	0.328	0.505
GFT	—	0.932	1.237	0.573	0.359	5.686	0.301	0.362	—	—	—
GFT+VAR	—	0.328	1.332	0.163	0.070	0.751	0.161	0.387	—	—	—
naive	0.231	0.242	0.961	0.179	0.064	0.317	0.182	0.400	0.090	0.231	0.669
**MAE**											
ARGO2	**0.224**	**0.229**	**0.509**	**0.251**	**0.156**	**0.295**	**0.202**	**0.283**	**0.186**	**0.277**	**0.344**
VAR	0.256	0.257	0.576	0.260	0.179	0.346	0.251	0.313	0.248	0.324	0.431
GFT	—	0.521	0.696	0.556	0.490	1.454	0.411	0.368	—	—	—
GFT+VAR	—	0.290	0.681	0.275	0.178	0.447	0.259	0.391	—	—	—
naive	0.268	0.271	0.636	0.289	0.182	0.355	0.268	0.372	0.230	0.327	0.519
**MAPE**											
ARGO2	**0.155**	**0.158**	**0.206**	0.140	**0.108**	**0.120**	**0.110**	**0.115**	**0.112**	**0.132**	**0.104**
VAR	0.164	0.166	0.230	**0.128**	0.123	0.147	0.130	0.125	0.145	0.146	0.121
GFT	—	0.354	0.253	0.270	0.347	0.626	0.260	0.162	—	—	—
GFT+VAR	—	0.175	0.246	0.130	0.120	0.171	0.140	0.163	—	—	—
naive	0.161	0.163	0.237	0.142	0.124	0.143	0.137	0.140	0.133	0.149	0.141
**Correlation**											
ARGO2	**0.963**	**0.954**	0.936	**0.951**	**0.825**	**0.935**	**0.938**	**0.952**	**0.934**	**0.938**	**0.974**
VAR	0.951	0.944	0.924	0.943	0.793	0.934	0.914	0.941	0.882	0.923	0.961
GFT	—	0.833	**0.946**	0.934	0.778	0.905	0.936	0.947	—	—	—
GFT+VAR	—	0.930	0.914	0.934	0.817	0.895	0.931	0.935	—	—	—
naive	0.942	0.933	0.902	0.928	0.791	0.914	0.895	0.908	0.897	0.912	0.937

The evaluation is based on the average of ten US HHS regions in multiple periods and multiple metrics, respectively. The MSE, MAE, MAPE and correlation are reported, comparing the estimates by different methods to CDC’s reported %ILI (the prediction target) over each period. The method with best performance is highlighted in boldface for each metric in each period. Methods considered here include ARGO2, VAR, GFT, GFT+VAR, and the naive method. All comparisons are conducted on the original scale of CDC’s %ILI. The whole period is March 29, 2009 to March 17, 2018. “2009–2015” is March 29, 2009 to August 15, 2015 following GFT’s availability. Columns 4 to 12 correspond to the 2009 off-season H1N1 outbreak, and every post-2009 regular flu reason (week 40 to week 20 next year, defined by CDC’s Morbidity and Mortality Weekly Report, 17′–18′ season up to March 17, 2018). Note that 2017–2018 is the validation period as the methodology of ARGO2 was frozen on December 26, 2016. All methods and metrics are defined in Methods section.

Compared to the benchmark methods, ARGO2’s advantages are apparent. Autoregression-based methods, VAR and GFT + VAR, hardly outperform the naive method. GFT performs rather poorly. It is worth noting that systematically outperforming the naive method for regional %ILI estimation is not easy, as shown in Supplementary Table S13, where most methods did worse than the naive method in 2015–2016 CDC’s Epidemic Prediction Initiative. When we apply ARGO2 to the data for the 2015–2016 CDC’s Epidemic Prediction Initiative, we see that ARGO2 is the only method that uniformly outperforms the naive method across all ten US HHS regions (see Supplementary Information for more details).

ARGO2 also shows the most consistent error reduction across all periods, notably during the H1N1 outbreak when regular flu seasonality did not hold (see Supplementary Table S16). Such consistency indicates the reliability of ARGO2 for accurate flu tracking in response to potential variations of flu epidemics. Particularly, for the flu outbreak during the 2017–2018 season, which was after the methodology of ARGO2 was frozen in December 2016, ARGO2 still maintains superior performance (MSE = 0.278, 41.5% relative to the naive method). This validates the robustness and flexibility of ARGO2, showcasing its capability to generate accurate estimates for irregular flu epidemics.

In addition to the weekly estimates, ARGO2 also gives confidence intervals. Table 2 and Supplementary Figs S14–S23 show the coverage of the confidence intervals for the ten US HHS regions. The nominal 95% confidence interval has an actual 94.96% coverage on average, suggesting that our confidence intervals closely measure the accuracy of our weekly estimates.

**Table 2:** Actual coverage of confidence intervals by ARGO2 for ten US HHS regions.

Region 1	Region 2	Region 3	Region 4	Region 5	Region 6	Region 7	Region 8	Region 9	Region 10
93.61%	92.63%	94.59%	95.33%	96.56%	94.10%	96.81%	95.58%	93.86%	96.56%

The coverage is for 95% nominal confidence level. The average coverage over the ten US HHS regions is 94.96%. Supplementary Figs S14–S23 plot the coverage in detail.

Detailed results of each of the ten US HHS regions (Supplementary Tables S1–S10 and Supplementary Figs S4–S13) show that ARGO2 gives rather robust estimation across various geographical regions (i.e., consistent error reduction across all time periods and regions). Across all regions, ARGO2 reaches at least 20% error reduction in MSE, and close to 10% in MAE compared with the naive method for the whole period, and it maintains the lead over all other benchmark methods. Breaking down different periods, ARGO2 holds the lead in most of the cases (about 75%). We also observe that Region 5 (containing US states IL, IN, MI, MN, OH, WI) gives the highest error reduction in the whole period (relative MSE = 0.487, relative MAE = 0.722 to naive), which may be attributed to the high correlation of Region 5 with neighboring regions (as shown in Fig. 1). This finding is consistent with^28^ and^29^, where the authors concluded that a network model is best in structurally central regions that have strong connections with neighboring regions.

## Discussion

ARGO2 efficiently combines multi-source, multi-resolution information and provides accurate, reliable, real-time flu tracking at the regional level. ARGO2 aggregates publicly available search data from Google at different resolutions (both national and regional) and traditional influenza surveillance data from the CDC. ARGO2 also incorporates cross-regional and temporal correlation of influenza activities for accurate estimation. Across all the regions, ARGO2 outperforms most previously available Internet-search-based regional influenza tracking methods. Such high-precision, regionally differential surveillance information, as provided by ARGO2, enables public health officials to make timely decisions regarding the changes in flu epidemics and to optimize the allocations of medical and personnel resources across the nation. Furthermore, ARGO2 also constructs confidence intervals as a measure of reliability for its weekly %ILI estimates. The reliable estimation by ARGO2 gives public health agencies and health care providers a head start in response planning for potential flu emergencies or outbreaks in the future.

The challenges of digital flu detection at the regional level include three important components: (1) Regional Internet-derived data are often of inferior quality to the national counterparts, typically containing weaker signals. For example, Google Trends search frequency data are much sparser (i.e., have more zero counts) at the regional level than at the national level (as illustrated in Supplementary Fig. S1). (2) The correlation structure of regional %ILI is complex and time-heterogeneous, which requires careful modeling efforts; see Fig. 1, which illustrates the strong spatial correlation across US HHS regions. (3) CDC’s regional ILI report is based on surveys with much smaller sample sizes than the national level, making CDC’s regional %ILI noisier than its national counterpart. Here we discuss in detail how ARGO2 address these challenges to achieve improved estimation accuracy.

In contrast to the low-resolution national level data, we note that the quality of the publicly available high-resolution Google Trends regional data is not as satisfactory. The high sparsity of Google Trends data (i.e., much more zero entries) for the majority of the search terms observed at the regional level (see Supplementary Fig. S1) severely impacts the contribution from Google search data to the %ILI estimation. We address this issue in our method by adopting an *L*_1_ penalty in step one to filter out low-quality sparse terms that correlate poorly with CDC’s historical %ILI.

In addition to the incorporation of Google search data, another key contributor to the improved accuracy of our estimation is the aggregation of multi-source, multi-resolution information. We include (i) the national baseline %ILI estimates (obtained from the ARGO method^25)^ based on the Google search data at the national level, (ii) Google search information at the regional level, and (iii) the region-specific %ILI time-series pattern and variation by considering the (time-series) increments of %ILI at the regional level. The national level %ILI estimates by ARGO capture not only the general average trends of flu activities, but also the seasonality.

We model the cross-regional and cross-resolution dependence through structuring the covariance matrix of the (time-series) %ILI increments, which effectively enhances the accuracy and efficiency in estimating the large covariance matrix with limited data in the two-year sliding window. The cross-correlation on the time-series increments captures the geographic spread of the flu and aggregates regional connectivity factors such as public transportation, geographic proximity, climatic patterns, and vaccination coverage. In fact, the cross-regional covariance matrices identified by ARGO2 (Supplementary Fig. S2) indicate stronger connectivity among central regions and northeastern regions, which agrees with the “regional clusters” found in^29^.

One of the limitations for our analysis is using CDC’s %ILI as our prediction target. We note that the reported %ILI is only a proxy for the actual flu incidence in the population. In fact, due to the reduced sample sizes, CDC’s reported %ILI at the regional level is even more susceptible to sampling bias, for instance, from enrolled health care providers in CDC’s ILINet or patients’ willingness to visit the providers. Nevertheless, despite the limitation of CDC’s %ILI itself, accurate estimation of CDC’s %ILI at the regional level can still help public health officials allocate resources in preparation for potential surges of patient visits to healthcare facilities^13,31,32^.

Another limitation of ARGO2 is for tracking rare events at the regional level. At the national level, the Google search data have low sparsity (i.e., few zero entries) and have been successfully utilized to track rare diseases such as Dengue fever in tropical countries^33,34^. However, the high sparsity (i.e., much more zero entries) in Google search data at the regional level (see Supplementary Fig. S1) could limit the usage of ARGO2 for regional rare disease tracking. A possible solution is to further extend ARGO2 by embracing alternative data sources such as electronic health records or Twitter text data.

The ARGO2 procedure has general applicability. The separation of the two steps offers the flexibility to substitute the first step (currently obtained from using Google Trends data) with the output from other models or data sources, while preserving the second step’s capability of multi-resolution spatial-temporal boosting. A wide variety of digital disease detection methods, including a Wikipedia-based mechanistic epidemic model^35^, a Google Trends-based Gaussian Process estimator^23^, a Twitter-based linear estimator^36^, a Twitter-based word-embedded nonlinear estimator^24^, or the ensemble of these estimators^37^, can be fitted into the cross-regional boosting step of ARGO2. The first step of ARGO2 can also incorporate other potentially relevant factors/predictors (e.g., weather) into the prediction model. In addition, such incorporation of multiple sources of information can potentially further extend ARGO2 to track other rare events (e.g. epidemics of rare diseases). The ARGO2 framework could be readily adapted to various spatial and temporal scales for tracking or forecasting other diseases and public health/social events that leave footprints on Internet users’ online records.

## Methods

### Google data

Google Trends is publicly available at trends.google.com. For a search query term specified by a user, Google Trends gives an integer valued (weekly) time series ranging from 0 to 100, which corresponds to the search intensity of the query term, where 100 represents the highest historical search volume. The publicly-available integer-valued time series is based on sampling Google’s raw search records. For benchmarking, we also downloaded the discontinued Google Flu Trends (GFT) data (www.google.org/flutrends/about/data/flu/us/data.txt). GFT has regional level prediction for %ILI from September 28, 2003 to August 15, 2015. GFT was discontinued in the week ending August 15, 2015.

The search query terms that we use are identified from Google Correlate (www.google.com/trends/correlate), which gives the most highly correlated search terms with a time series supplied by a user (detailed in Supplementary Information). Supplementary Tables S11 and S12 in Supplementary Information list these search terms.

### Aggregation of state-level Google search data

Google Trends only provides state-level, rather than regional, search frequency data. We use a simple approach to estimate the regional search frequency based on aggregation of state level data. Specifically, to estimate each search query term’s regional frequency, we use a weighted average of the integer-valued time series returned by Google Trends for each of the states in that region, where the weights are proportional to the state population according to the 2010 US census.

### CDC’s ILINet data

CDC’s ILINet invites more than 2,800 enrolled outpatient health care providers around the US to report the number of patients with influenza-like illness (defined as fever and cough and/or a sore throat without a known cause other than influenza) together with the total number of all patients seen (www.cdc.gov/flu/weekly/overview.htm). The reports from health care providers are later aggregated at the US HHS regional level as well as at the national level. Each Friday, CDC releases a weekly ILI report for the previous week, where the %ILI are reported at both the US national level and at the HHS region level. The initial publication is subject to later revision as CDC receives updated data or late submissions from the health care providers. Consequently, CDC’s ILI report always lags behind real-time for 1 to 2 weeks and is less accurate for more recent weeks. CDC’s weekly %ILI are publicly available at gis.cdc.gov/grasp/fluview/fluportaldashboard.html.

### The model of ARGO2

#### Step One: extracting Internet search information

At this step, we obtain a preliminary estimate for the regional level %ILI based completely on region-wise Google search data. This preliminary estimate will be the input for the second step. For a given region *m*, *m* = 1, …, 10, let *x*_*i*,*t*,*m*_ be the logarithm of the aggregated region-level Google search frequency of search term *i* at week *t*; let *y*_*t*,*m*_ be the logit-transformation of CDC’s (weighted) %ILI at time *t* for region *m*. At week *T*, owing to the time delay of CDC’s ILI reports, *y*_*t*,*m*_ would be available only up to *T*−1, whereas the variables *x*_*i*,*t*,*m*_ are available up to time *T*.

For each region *m*, to estimate *y*_*T*,*m*_, we use a *L*_1_ regularized linear estimator in the first step:

$${\hat{y}}_{T,m}={\hat{\beta }}_{0,m}+{{\bf{X}}}_{T,m}^{T}{\hat{{\boldsymbol{\beta }}}}_{m},$$

where the vector **X**_*T*,*m*_ = (*x*_*i*,*T*,*m*_), and $({\hat{\beta }}_{0,m},{\hat{{\boldsymbol{\beta }}}}_{m})$ is obtained via

$$argmi{n}_{{\beta }_{0,m},{{\boldsymbol{\beta }}}_{m}}\sum _{t=T-N}^{T-1}{({y}_{t,m}-{\beta }_{0,m}-{{\bf{X}}}_{t,m}^{T}{{\boldsymbol{\beta }}}_{m})}^{2}+\lambda {\Vert {{\boldsymbol{\beta }}}_{m}\Vert }_{1}.$$

*N* in the above equation is the training window. We set *N* = 104, i.e., a two-year window. The use of this moving window, as recommended in previous studies^25,30^, is to account for changes in people’s search patterns as well as changes in the search engine itself. The *L*_1_ penalty^38^ adds sparsity in estimated coefficients ${\hat{{\boldsymbol{\beta }}}}_{m}$ for all input search query terms. Due to the penalty, noisy terms with zero coefficients are eliminated from the model, and the most relevant search terms with non-zero coefficients are automatically selected. We set *λ* through cross-validation. In addition, we also obtain an accurate estimate ${\hat{y}}_{T}^{nat}$ for the national %ILI by using the ARGO method^25^, which uses national level Google search data.

#### Step Two: cross-regional boosting

The second step aggregates multi-resolution and time series information to boost the regional-level estimation. It derives the best linear predictor based on the time series increments of %ILI, the preliminary regional %ILI estimates, and the accurate national %ILI estimates, while also accounting for cross-source, cross-region and temporal correlations.

Let ***p***_*t*_ = (*p*_*t*,1_, …, *p*_*t*,10_)^*T*^ denote CDC’s %ILI of the ten US HHS regions; they are related to *y*_*t*,*m*_ through the inverse logit transformation *p*_*t*,*m*_ = exp(*y*_*t*,*m*_)/(1 + exp(*y*_*t*,*m*_)). Our preliminary estimate for ***p***_*t*_ from the first step is ${\hat{{\boldsymbol{p}}}}_{t}^{GT}={({\hat{p}}_{t,1},\ldots ,{\hat{p}}_{t,10})}^{T}$, where ${\hat{p}}_{t,m}=\exp ({\hat{y}}_{t,m})/(1+\exp ({\hat{y}}_{t,m}))$. Our estimate of the national %ILI from the first step is ${\hat{p}}_{t}^{nat}=\exp ({\hat{y}}_{t}^{nat})/(1+\exp ({\hat{y}}_{t}^{nat}))$. Let the boldfaced ${\hat{{\boldsymbol{p}}}}_{t}^{nat}$ denote the length-10 vector ${\hat{{\boldsymbol{p}}}}_{t}^{nat}={({\hat{p}}_{t}^{nat},\ldots ,{\hat{p}}_{t}^{nat})}^{T}$.

Estimating ***p***_*t*_ is equivalent to estimating the time series increment Δ***p***_*t*_ = ***p***_*t*_ − ***p***_*t*−1_. We denote **Z**_*t*_ = *Δ****p***_*t*_ for notational simplicity. For the estimation of **Z**_*t*_, we want to incorporate the cross-regional, cross-source correlations. Three predictors for **Z**_*t*_ after the first step are thereby included: (i) **Z**_*t*−1_ = *Δ****p***_*t*−1_, (ii) ${\hat{{\boldsymbol{p}}}}_{t}^{GT}-{{\boldsymbol{p}}}_{t-1}$, and (iii) ${\hat{{\boldsymbol{p}}}}_{t}^{nat}-{{\boldsymbol{p}}}_{t-1}$; they represent time series information, information from the regional level Google search, and the information from the national level Google search, respectively. Let **W**_*t*_ denote the collection of these three predictors: ${{\bf{W}}}_{t}={({{\bf{Z}}}_{t-1}^{T},{({\hat{{\boldsymbol{p}}}}_{t}^{GT}-{{\boldsymbol{p}}}_{t-1})}^{T},{({\hat{{\boldsymbol{p}}}}_{t}^{nat}-{{\boldsymbol{p}}}_{t-1})}^{T})}^{T}$. To combine these predictors, we use the best linear predictor formed by them:

1 $${\hat{{\bf{Z}}}}_{t}={{\boldsymbol{\mu }}}_{Z}+{{\rm{\Sigma }}}_{ZW}\,{{\rm{\Sigma }}}_{WW}^{-1}\,({{\bf{W}}}_{t}-{{\boldsymbol{\mu }}}_{W}),$$

where *μ*_*Z*_ and *μ*_*W*_ are the mean vectors of **Z** and **W** respectively, and *Σ*_*ZZ*_, *Σ*_*ZW*_ and *Σ*_*WW*_ are the covariance matrices of and between **Z** and **W**. The best linear predictor gives the optimal way to linearly combine the three predictors to form a new one. It also provides the variance estimate as

2 $${\rm{V}}{\rm{a}}{\rm{r}}({{\bf{Z}}}_{{\boldsymbol{t}}}|{{\bf{W}}}_{{\boldsymbol{t}}})={{\rm{\Sigma }}}_{ZZ}-{{\rm{\Sigma }}}_{ZW}{{\rm{\Sigma }}}_{WW}^{-1}{{\rm{\Sigma }}}_{WZ}.$$

Consistent with the first step, we adopt a sliding two-year training window to estimate ***μ***_*Z*_, ***μ***_*W*_, *Σ*_*ZZ*_, *Σ*_*ZW*_ and *Σ*_*WW*_ in Equation (1). For *μ*_*Z*_ and *μ*_*W*_, we use the empirical means of the corresponding variables as the estimators. However, for the covariance matrices, due to their large sizes – 30 × 30 for *Σ*_*WW*_ and 10 × 30 for *Σ*_*ZW*_ – and the small number of observations – 104 weekly training data points in the two-year window, we need to structure the covariance matrices for reliable estimation. We assume the following structure:

The covariance between the time series increments satisfies *cov*(**Z**_*t*_) = *cov*(**Z**_*t*−1_) = *Σ*_*ZZ*_ and *cov*(**Z**_*t*_, **Z**_*t*−1_) = *ρ*Σ_*ZZ*_, where 0 < *ρ* < 1: This essentially assumes that the time series increments are stationary and have a stable autocorrelation across time and region. We could more generally assume *cov*(**Z**_*t*_, **Z**_*t*−1_) is *Σ*_*ZZ*_ multiplied by a diagonal matrix. However, our numerical investigation suggested that the additional flexibility would not help in the estimation performance, mainly due to the substantial extra variance incurred by these additional parameters. Therefore, we keep the simple assumption of *cov*(**Z**_*t*_, **Z**_*t*−1_) = *ρΣ*_*ZZ*_.
(i) Independence between the time series increment and the estimation error of the first-step regional estimates, i.e., ${{\bf{Z}}}_{t}\perp ({\hat{{\boldsymbol{p}}}}_{t}^{GT}-{{\boldsymbol{p}}}_{t})$, and (ii) cross-regional independence between the estimation error of the first-step regional estimates: (i) and (ii) are based on the fact that the first-step preliminary regional estimates are carried out separately for each region based solely on regional Google search information of that region alone. Mathematically, (i) and (ii) imply that $D=cov({\hat{{\boldsymbol{p}}}}_{t}^{GT}-{{\boldsymbol{p}}}_{t})$ is diagonal, $cov({{\bf{Z}}}_{t},{\hat{{\boldsymbol{p}}}}_{t}^{GT}-{{\boldsymbol{p}}}_{t-1})={\Sigma }_{ZZ}$, and $cov({\hat{{\boldsymbol{p}}}}_{t}^{GT}-{{\boldsymbol{p}}}_{t-1})={\Sigma }_{ZZ}+D$.
Independence between the time series increment and the regional deviates from national baseline, i.e., ${{\bf{Z}}}_{t}\perp ({\hat{{\boldsymbol{p}}}}_{t}^{nat}-{{\boldsymbol{p}}}_{t})$: Thereby, we have $cov({{\bf{Z}}}_{t},{\hat{{\boldsymbol{p}}}}_{t}^{nat}-{{\boldsymbol{p}}}_{t-1})={\Sigma }_{ZZ}$ and $cov({\hat{{\boldsymbol{p}}}}_{t}^{nat}-{{\boldsymbol{p}}}_{t-1})={\Sigma }_{ZZ}+{\Sigma }^{nat}$, where ${{\rm{\Sigma }}}^{nat}=cov({\hat{{\boldsymbol{p}}}}_{t}^{nat}-{{\boldsymbol{p}}}_{t})$.
Independence between the errors from two separate sources of estimation, i.e., (${\hat{{\boldsymbol{p}}}}_{t}^{GT}-{{\boldsymbol{p}}}_{t})\perp ({\hat{{\boldsymbol{p}}}}_{t}^{nat}-{{\boldsymbol{p}}}_{t})$: Mathematically, this implies $cov({\hat{{\boldsymbol{p}}}}_{t}^{GT}-{{\boldsymbol{p}}}_{t-1},{\hat{{\boldsymbol{p}}}}_{t}^{nat}-{{\boldsymbol{p}}}_{t-1})={\Sigma }_{ZZ}$.

The covariance matrices are thereby simplified as:

$$\begin{array}{ccc}{{\rm{\Sigma }}}_{ZW} & = & (\begin{array}{ccc}\rho {{\rm{\Sigma }}}_{ZZ} & {{\rm{\Sigma }}}_{ZZ} & {{\rm{\Sigma }}}_{ZZ}\end{array}),\\ {{\rm{\Sigma }}}_{WW} & = & (\begin{array}{ccc}{{\rm{\Sigma }}}_{ZZ} & \rho {{\rm{\Sigma }}}_{ZZ} & \rho {{\rm{\Sigma }}}_{ZZ}\\ \rho {{\rm{\Sigma }}}_{ZZ} & {{\rm{\Sigma }}}_{ZZ}+D & {{\rm{\Sigma }}}_{ZZ}\\ \rho {{\rm{\Sigma }}}_{ZZ} & {{\rm{\Sigma }}}_{ZZ} & {{\rm{\Sigma }}}_{ZZ}+{{\rm{\Sigma }}}^{nat}\end{array}).\end{array}$$

The assumptions made are supported by empirical evidence: (1) close agreement is observed between our estimated structural matrix and the empirical one (Supplementary Fig. S2), and (2) stationary bootstrap indicates statistical acceptance of the null hypothesis of our assumed covariance structure (Supplementary Fig. S3).

To further control the estimation stability, we incorporate a ridge-regression^39^ inspired shrinkage to the linear predictor in Equation (1), replacing the 40 × 40 joint covariance matrix of ${({{\bf{Z}}}_{t}^{T},{{\bf{W}}}_{t}^{T})}^{T}$ by the average of the structured covariance matrix and its empirical diagonal. Effectively, in Equation (1), *Σ*_*ZW*_ is replaced by $\frac{1}{2}{\Sigma }_{ZW}$, and *Σ*_*WW*_ is replaced by $(\frac{1}{2}{\Sigma }_{WW}+\frac{1}{2}{D}_{WW})$, where *D*_*WW*_ is the diagonal of the empirical covariance of **W**_*t*_:

$${\hat{{\bf{Z}}}}_{t}={{\boldsymbol{\mu }}}_{Z}+\frac{1}{2}{\Sigma }_{ZW}{(\frac{1}{2}{\Sigma }_{WW}+\frac{1}{2}{D}_{WW})}^{-1}({{\bf{W}}}_{t}-{{\boldsymbol{\mu }}}_{W}).$$

Similarly, we update Equation (2) with above substitution as well:

$${\rm{V}}{\rm{a}}{\rm{r}}({{\bf{Z}}}_{t}|{{\bf{W}}}_{t})={\Sigma }_{ZZ}-\frac{1}{2}{\Sigma }_{ZW}{(\frac{1}{2}{\Sigma }_{WW}+\frac{1}{2}{D}_{WW})}^{-1}\frac{1}{2}{\Sigma }_{WZ}.$$

*Σ*_*ZZ*_, *Σ*^*nat*^, *D*, and *D*_*WW*_ are estimated by the corresponding sample covariance from the data in the most recent 2-year training window; *ρ* is estimated by minimizing the Frobenius norm (*L*_2_ distance) between the empirical correlation and structured correlation.

Our final regional %ILI estimate for week *T* after step two is:

$${\hat{{\boldsymbol{p}}}}_{T}={{\boldsymbol{p}}}_{T-1}+{\hat{{\boldsymbol{\mu }}}}_{Z}+{\hat{\Sigma }}_{ZW}{({\hat{\Sigma }}_{WW}+{\hat{D}}_{WW})}^{-1}({{\bf{W}}}_{T}-{\hat{{\boldsymbol{\mu }}}}_{W}),$$

with a corresponding 95% confidence interval as:

$$[{\hat{{\boldsymbol{p}}}}_{T}\pm 1.96\times \sqrt{{\hat{\Sigma }}_{ZZ}-\frac{1}{2}{\hat{\Sigma }}_{ZW}{({\hat{\Sigma }}_{WW}+{\hat{D}}_{WW})}^{-1}{\hat{\Sigma }}_{WZ}}].$$

### Accuracy metrics

The MSE, MAE, MAPE and correlation of estimator $\tilde{p}$ to the target *p* for US HHS region *i* are: ${\rm{MSE}}({\tilde{p}}_{i},{p}_{i})=\frac{1}{n}\sum _{t=1}^{n}\,{({\tilde{p}}_{i,t}-{p}_{i,t})}^{2}$; ${\rm{MAE}}({\tilde{p}}_{i},{p}_{i})=\frac{1}{n}\sum _{t=1}^{n}\,|{\tilde{p}}_{i,t}-{p}_{i,t}|$; ${\rm{MAPE}}({\tilde{p}}_{i},{p}_{i})=\frac{1}{n}\sum _{t=1}^{n}\,|{\tilde{p}}_{i,t}-{p}_{i,t}|/{p}_{i,t}$. The correlation is the Pearson correlation between *p*_*i*,*t*_ and ${\mathop{p}\limits^{ \sim }}_{i,t}$ across index *t*. Note that all the metrics are computed on the original %ILI scale.

### Benchmark methods

We compare ARGO2 with benchmark methods including VAR, GFT, GFT + VAR, and naive estimation. VAR is the lag-1 vector autoregressive model (VAR-1 model) on the 10-region multivariate logit-transformed %ILI, i.e. ${{\boldsymbol{y}}}_{t}={\boldsymbol{c}}+{A}_{1}{{\boldsymbol{y}}}_{t-1}+{{\boldsymbol{\epsilon }}}_{t}$. GFT is the GFT estimates produced by Google (discontinued in the week ending on August 15, 2015). GFT + VAR is the lag-1 vector autoregressive model with GFT estimate as exogenous variable, i.e., ${{\boldsymbol{y}}}_{t}={\boldsymbol{c}}+{A}_{1}{{\boldsymbol{y}}}_{t-1}+B{{\boldsymbol{y}}}_{t}^{GFT}+{{\boldsymbol{\epsilon }}}_{t}$, where ${{\boldsymbol{y}}}_{t}^{GFT}$ is the logit-transformed GFT estimates. The naive method uses the previous week’s CDC observation as the estimate for the current week.

## Supplementary information

Supplementary Information
