# Supplementary material for: Accurate regional influenza epidemics tracking using Internet search data
Source: Sci Rep. 2019 Mar 27;9:5238. doi: 10.1038/s41598-019-41559-6 (PMC6437143; doi:10.1038/s41598-019-41559-6)
Supplement: Supplementary file 1 — Supplementary Information [file 41598_2019_41559_MOESM1_ESM.pdf]

Supplementary Information for

Accurate regional influenza epidemics tracking using Internet search data

Shaoyang Ning, Shihao Yang, and S. C. Kou

Correspondence to: kou@stat.harvard.edu

This PDF file includes:

Supplementary Text
Supplementary Figs. S1 to S23
Supplementary Tables S1 to S16

Supplementary Text

The Supplementary Information is organized as follows. First, the US national Google Trends data and regional Google Trends data are compared, and we show that the strong sparsity of the regional data indicates weaker input signal at the regional level. Second, detailed region-by-region estimation results are presented. Third, search query terms used in ARGO2 are listed. Fourth, performance of the methods in CDC's 2015-2016 Epidemic Prediction Initiative (FluSight) are presented. Fifth, the Relative Efficiencies comparing benchmark methods to ARGO2 are reported, along with the corresponding confidence intervals. Additionally, the statistical significance of ARGO2's improvement over benchmark methods is validated. Sixth, the motivations of the ARGO2 model are discussed. Seventh, additional evaluation metrics, including metrics on over-estimation and under-estimation, are provided.

Sparsity of regional Google Trends data

Google Trends provides search query frequency data at the US state and national levels. Google Trends data are generated by sampling users' query logs. Due to the difference in the samples and the sample sizes when Google does the sampling and other undisclosed details, the reported Google Trends data at the national level are *not* a simple weighted average of the corresponding reported state-level Google Trends data.

Supplementary Fig. S1 illustrates the lower quality of the regional Google Trends data. In contrast to the Google Trends data at the national level, the majority of the regional heat map is in white, corresponding to zero entries in the observed search frequencies (national sparsity rate 12.8% vs. regional 60.0%). Such sparsity adds substantial difficulty to extracting information from Google search data for accurate %ILI estimation. This sparsity also explains why directly applying national estimation methods to regional Google Trends data tends to fail.

Detailed Results Region by Region

Supplementary Tables S1 – S10 report the detailed estimation results for each of the ten US HHS regions. The ***relative*** MSE, MAE, and MAPE to the naive methods (i.e., the ratio of the measures between a specific method and the naive method) and the correlation are reported, with the best performance (in each time period for each metric) in boldface and the original metrics for the naive method in parentheses. We compare the ARGO2 estimates with benchmark methods, including VAR, GFT, GFT+VAR, and the naive method. All comparisons are conducted on the original scale of %ILI. The time periods in the table are: "whole period" (from March 29, 2009 to March 17, 2018); "2009-2015" (from March 29, 2009 to August 15, 2015 when GFT estimates are available); "off-season H1N1 outbreak period" (from March 29, 2009 to December 27, 2009); and the other columns are regular flu seasons (week 40 to week 20 next year, 17'-18' season up to March 17, 2018) after 2009. Supplementary Figs. S4–S13 plot the time series of the estimated %ILI in comparison with the CDC's reported %ILI, as well as the series of estimation errors, from March 29, 2009 to March 17, 2018. Supplementary Figs.

S14–S23 plot the coverage of 95% Confidence Interval constructed by ARGO2 in comparison to the actual CDC's reported %ILI (the prediction target).

Search query terms used in ARGO2

For the estimation before May 22, 2010, we use 70 query terms (listed in Supplementary Table S11). These query terms are identified by supplying CDC's national %ILI data from January 2004 to March 2009 to Google Correlate and then removing terms unrelated to flu. For estimation after May 22, 2010, we use 59 additional query terms (i.e., 129 terms in total). These additional 59 terms are identified by supplying CDC's national %ILI data from January 2004 to May 2010 to Google Correlate (listed in Supplementary Table S12).

Results from CDC's Epidemic Prediction Initiative

We show the results of the participants in the 2015-2016 CDC Epidemic Prediction Initiative (FluSight) for nowcasting CDC's (weighted) %ILI in the ten US HHS regions in Supplementary Table S13. The data are publicly available at https://github.com/cdcepi/FluSight-forecasts, under license Creative Commons Attribution 4.0. The true %ILI, i.e., the estimation target, is the *finalized* %ILI on the CDC's report (revealed weeks after the estimation period). In the table, we report the ***relative*** MSE of the participants' estimation to the naive method, i.e., the ratio between the MSE of each method and the MSE of the naive method. We report the overall relative errors by averaging over the ten regions as well as the relative errors for each region. The

naive estimate uses the previous week's %ILI on CDC's latest unrevised flu report available at the week of estimation (the CDC's report is subject to later revision) as the estimate for the current week. The methods submitted to the challenge include: 4Sight; ARETE; two methods from Columbia University (CU1, CU2, http://cpid.iri.columbia.edu); Delphi-Archefilter, Delphi-Epicast, Delphi-Stat(http://delphi.midas.cs.cmu.edu); a method from Iowa State University (ISU); JL; a method from Knowledge Based Systems, Inc. (KBSI1); Kernel of Truth (KOT, no regional estimates, http://reichlab.io); a method from MOBS-Lab (NEU, http://www.mobs-lab.org); a method from Predictive Science, Inc. (PSI, http://www.predsci.com/portal/home.php); a method from HumNat Lab (UMN, http://www.tc.umn.edu/~matteoc/Welcome.html). We also compared the results with ARGO2 and VAR. Notably, ARGO2 is the only method that uniformly outperforms the naive method across all ten regions.

Relative Efficiency

Supplementary Table S14 reports the Relative Efficiency of ARGO2 to other benchmark methods with the 95% confidence intervals. ARGO2 significantly outperforms all benchmark models considered in this study, as the 95% confidence intervals are all strictly above 1.

The Relative Efficiency is based on the average MSE of ten US HHS regions from method (a) to method (b). It is estimated as $\hat{e}\,(\widetilde{\mathbf{p}}^{(a)}, \widetilde{\mathbf{p}}^{(b)}) = \sum_{i=1}^{10} \text{MSE}\,(\widetilde{p}_i^{(b)}, p_i)/$

$\sum_{i=1}^{10}$ MSE $(\tilde{p}_i^{(a)}, p_i)$. The terms $\tilde{p}_i^{(a)}$ and $\tilde{p}_i^{(b)}$are %ILI estimators for region i by the two methods respectively, and the MSE of estimator $\tilde{p}$ to the target p is given by $\text{MSE}(\tilde{p}_i, p_i) = \frac{1}{n}\sum_{t=1}^{n}(\tilde{p}_{i,t} - p_{i,t})^2$. The 95% confidence interval (CI) is obtained by vector stationary bootstrap on the index t with mean block size 5 (equivalent to 1 month of data) [1]. We first obtain the basic bootstrap CI for logarithm of Relative Efficiency and then recover the original scale by exponentiation. The nonparametric vector stationary bootstrap controls for cross-region spatial correlation and for cross-time autocorrelation of the error residuals. The bootstrap procedure is robust to mean block size.

Motivation of the ARGO2 model

ARGO2 can be thought of as motivated by a hidden Markov structure on the three predictors: (i) changes in the %ILI $\Delta\mathbf{p}_t$, evolving according to a time series (e.g., autoregression), (ii) regional estimation $\hat{\mathbf{p}}_t^{GT}$ based on the regional Google search data, and (iii) national estimation $\hat{\mathbf{p}}_t^{nat}$ based on national Google search data. (ii) and (iii) are separately produced to estimate $\mathbf{p}_t$ from data at two different resolutions. The following diagram illustrates their relationship.

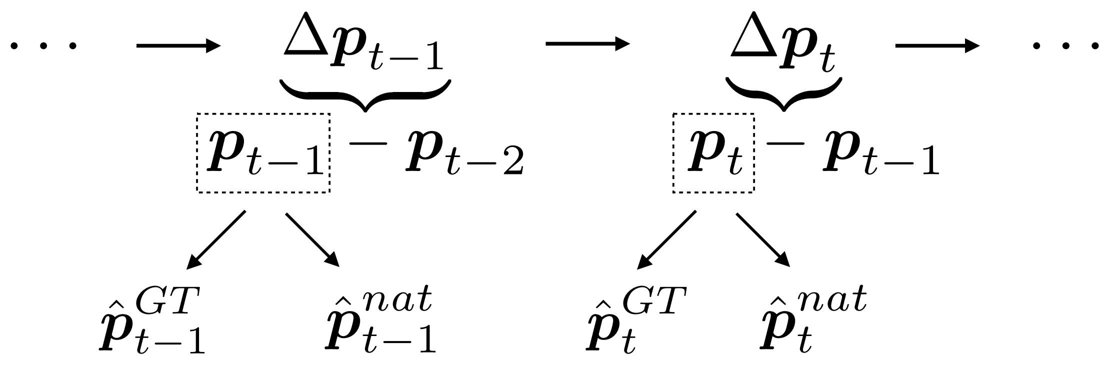

By modeling the joint covariance matrix of the three predictors and using the best linear predictor of them, we achieve better estimation efficiency compared to partial models, such as the linear regression model on each individual region. We are able to take full advantage of the correlation structure of the data. In fact, for most conventional regression models, such correlation structure is ignored, and each region has its own regression trained separately. Also, jointly modeling the covariance on the original level ($\mathbf{p}_t$) of %ILI as opposed to its logit-transformed version ($\mathbf{y}_t$) ensures the best linear predictor is optimal in mean squared error for estimating $\mathbf{Z}_t^T$.

Our assumed covariance structure is validated by statistical testing and empirical comparison (Supplementary Figs. S2 and S3). Supplementary Fig. S2 displays the 40×40 joint covariance and correlation matrices of $(\mathbf{Z}_t^T, \mathbf{W}_t^T)^T$. The left column plots the average of the structured covariance/correlation matrices under our assumptions 1-4, described in the Method section. The right column plots the average of all the empirical covariance/correlation matrices. All matrices are estimated from the two-year rolling window at each week from March 29, 2009 to October 1, 2016. The close agreement between the two columns further supports our assumptions 1- 4 on the covariance matrix structure.

We also validate our assumed covariance structure statistically based on a stationary bootstrap [1] on the time series $(\mathbf{p}_t, \hat{\mathbf{p}}_t^{GT}, \hat{\mathbf{p}}_t^{nat})$ with mean block size of 52. The element-

wise p-values for the structured covariance matrix are obtained by referring the structured covariance matrix to the bootstrapped distribution of the average empirical covariance matrix of $(\mathbf{Z}_t^T, \mathbf{W}_t^T)^T$. The average p-value is 0.29, indicating statistical acceptance of the null hypothesis of our assumed covariance structure (heat map of p-values shown in Supplementary Fig. S3).

Our choice to model the increments $\Delta\mathbf{p}_t$ of %ILI is based on the observation that the goodness of fit of the VAR model is better on $\Delta\mathbf{p}_t$ than on $\mathbf{p}_t$. In addition, the spatial spread of the flu corresponds to how many more people are infected by flu at a given week compared to the previous one, i.e., change in flu activity levels. Modeling on the increments also captures such intuition.

Finally, our inclusion of ridge-regression type shrinkage on the 40×40 joint covariance matrix of $(\mathbf{Z}_t^\intercal, \mathbf{W}_t^\intercal)^\intercal$ is motivated by the estimation improvement (in terms of mean squared error) achieved by ridge regression over ordinary linear regression.

Additional evaluation metrics

We also compared ARGO2 with benchmark methods using additional evaluation metrics: mean squared error on overestimation (MSE+), mean squared error on underestimation (MSE-), and bias (Bias). Suppose $\widetilde{p}_t$ is the estimator for the target ILI activity level p_t at time t. The metrics are then defined as follows: $\mathrm{MSE}_+(\widetilde{p}, p) = \frac{1}{\sum_{t=1}^{n} I_{\widetilde{p}_t > p_t}} \sum_{t=1}^{n} (\widetilde{p}_t -$

$p_t)^2 I_{\tilde{p}_t > p_t}$; $\text{MSE}_{-}(\tilde{p}, p) = \frac{1}{\sum_{t=1}^{n} I_{\tilde{p}_t < p_t}} \sum_{t=1}^{n} (\tilde{p}_t - p_t)^2 I_{\tilde{p}_t < p_t}$; $\text{Bias}(\tilde{p}, p) = \frac{1}{n} \sum_{t=1}^{n} (\tilde{p}_t - p_t)$.

We report the overall results in Supplementary Table S15, averaging over the ten US HHS regions. In general, ARGO2 still shows advantages in robustness and accuracy across these metrics. Notably, ARGO2 holds advantages in all of these additional metrics during the whole evaluation period (March 29, 2009 to March 17, 2018); when separating the MSE into over-estimation and under-estimation, ARGO2 is the only method that consistently outperforms the naive method in all seasons. In addition, ARGO2 outperforms all other methods uniformly in all periods in MSE+, with small lags behind the best numbers in MSE- and Bias. ARGO2 also maintains a relatively balanced performance between over-estimation and under-estimation over various seasons. Such balanced performance suggests the robustness of ARGO2 from another angle.

We also report in Table S16 the overall results regarding ***relative*** MSE, MAE and MAPE to the naive method, as a supplement to Table 1 (in original error metrics).

References

1. Politis, D. N. & White, H. Automatic Block-Length Selection for the Dependent Bootstrap. *Econom. Rev.* **23,** 53–70 (2004).

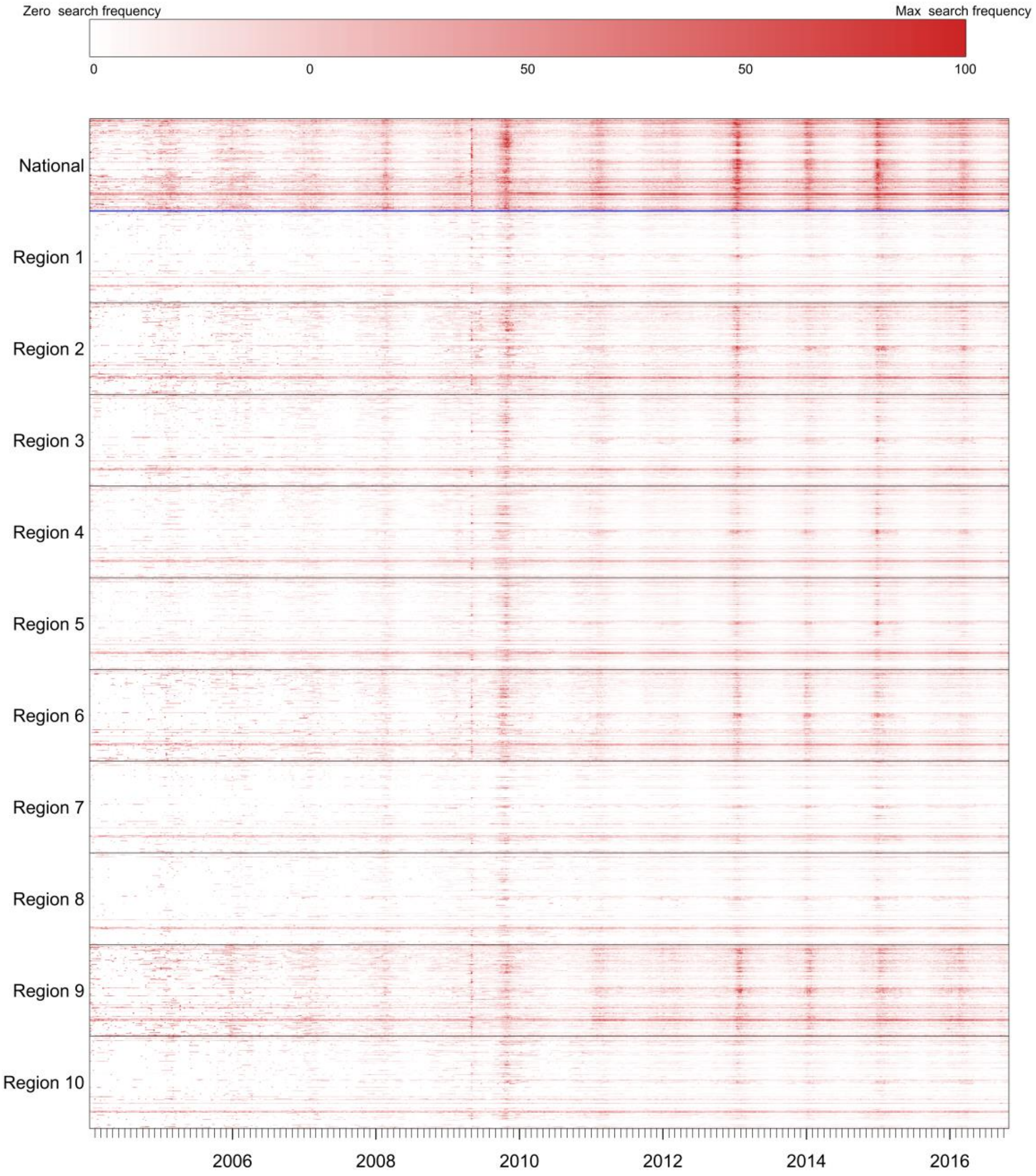

Supplementary Figure S1. National Google Trends data and Regional Google Trends (GT) data. The thick blue horizontal line separates national data from regional data. The thin black horizontal lines separate data of different regions. Each block consists of 129 query terms whose GT values across time are plotted as heat map with 0

being white and 100 being red. As shown in the figure, the sparsity of the regional GT data is much higher than that of the national GT data, indicating that regional GT data are of much lower quality than the national counterpart.

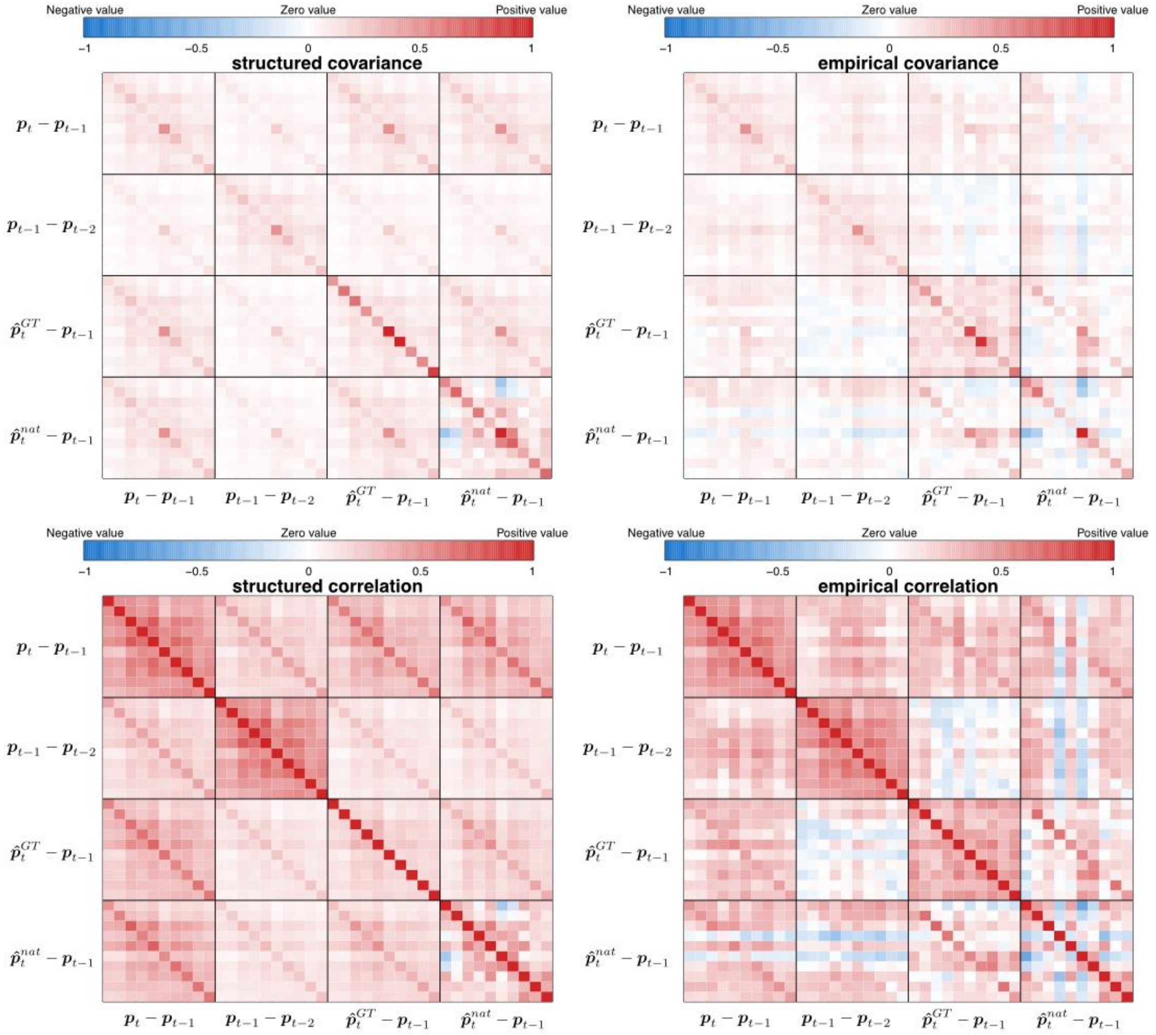

Supplementary Figure S2. Heat maps of the structured and empirical $\mathbf{40 \times 40}$ covariance and correlation matrices of $(\boldsymbol{Z_t^T}, \boldsymbol{W_t^T})^{\boldsymbol{T}}$. The left column is based on the average of all the structured covariance/correlation matrices obtained from the two-year training data at each week of evaluation from March 29, 2009 to October 1, 2016, and the right column is based on the average of all the empirical covariance/correlation matrices obtained from the two-year training data at each week of evaluation during the same evaluation period.

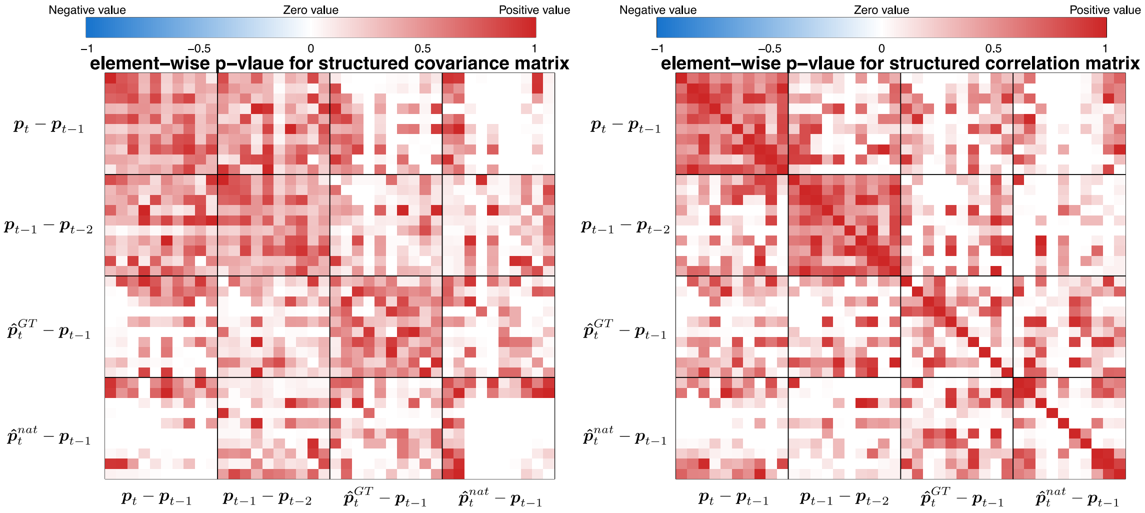

Supplementary Figure S3. Heat maps of the element-wise p-values for structured covariance matrix of $(\boldsymbol{Z}_t^T, \boldsymbol{W}_t^T)^T$. The element-wise p-values for the null hypothesis of structured covariance (left) and correlation (right) matrix based on stationary bootstrap are plotted. The average of these p-values is 0.29, indicating statistical acceptance of the null hypothesis of the assumed covariance structure.

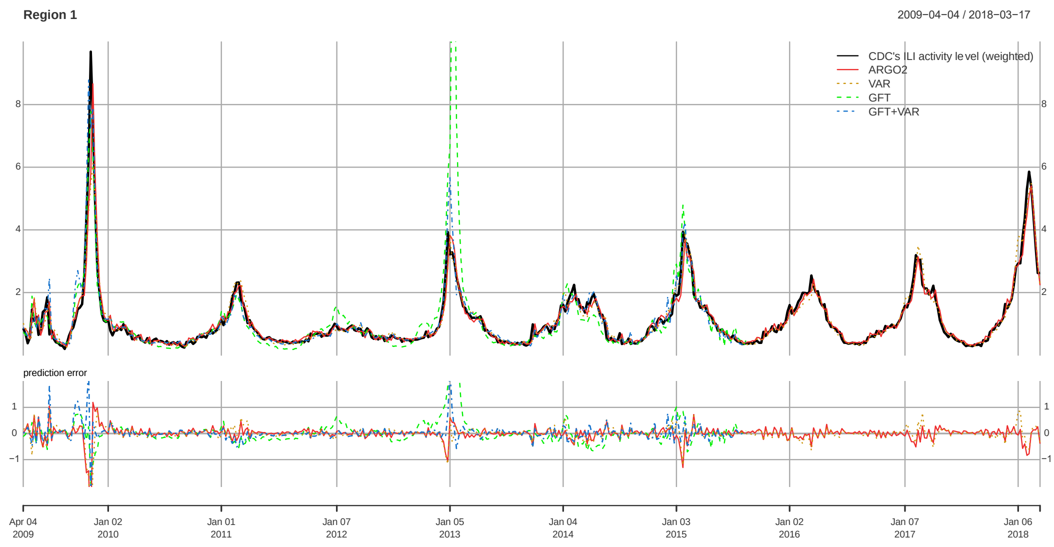

Supplementary Figure S4. Plots of the %ILI estimates (top) and the estimation errors (bottom) for Region 1. The evaluation period is from March 29, 2009 to March 17, 2018. Methods compared include ARGO2 (solid red), GFT (dashed green), VAR (dotted yellow) and GFT+VAR (dash-dotted blue), in contrast with CDC's weighted %ILI activity level (solid black).

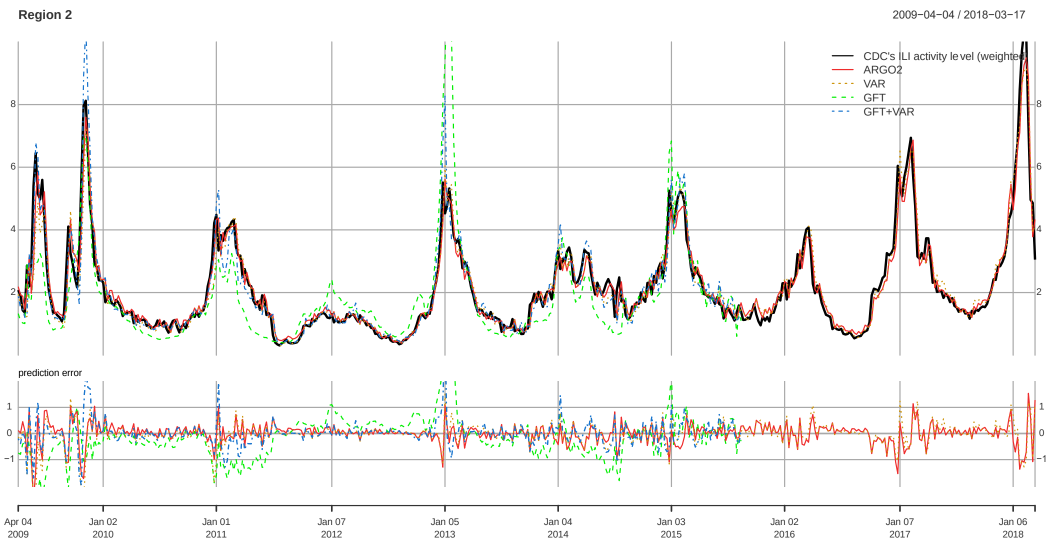

Supplementary Figure S5. Plots of the %ILI estimates (top) and the estimation errors (bottom) for Region 2. The evaluation period is from March 29, 2009 to March 17, 2018. Methods compared include ARGO2 (solid red), GFT (dashed green), VAR (dotted yellow) and GFT+VAR (dash-dotted blue), in contrast with CDC's weighted %ILI activity level (solid black).

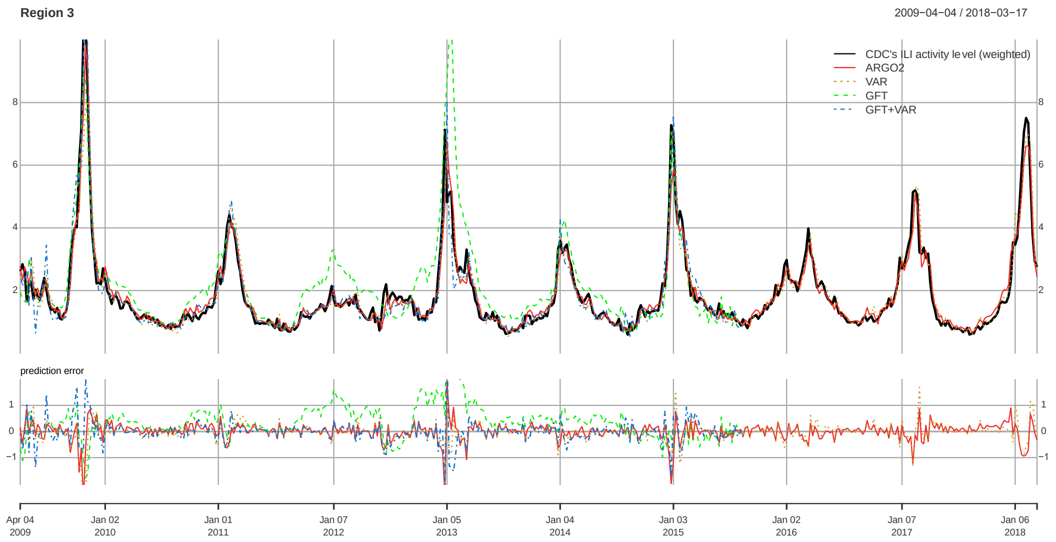

Supplementary Figure S6. Plots of the %ILI estimates (top) and the estimation errors (bottom) for Region 3. The evaluation period is from March 29, 2009 to March 17, 2018. Methods compared include ARGO2 (solid red), GFT (dashed green), VAR (dotted yellow) and GFT+VAR (dash-dotted blue), in contrast with CDC's weighted %ILI activity level (solid black).

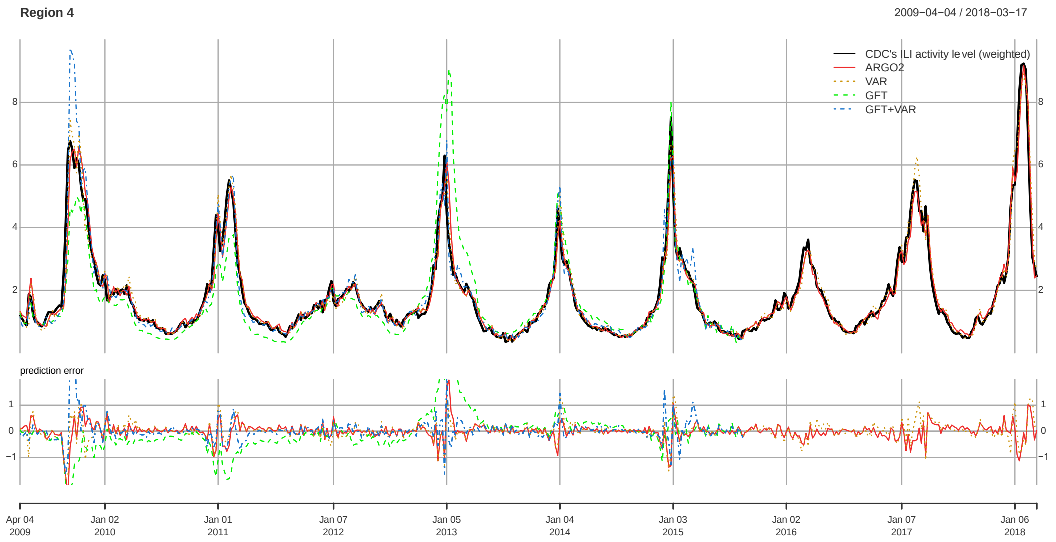

Supplementary Figure S7. Plots of the %ILI estimates (top) and the estimation errors (bottom) for Region 4. The evaluation period is from March 29, 2009 to March 17, 2018. Methods compared include ARGO2 (solid red), GFT (dashed green), VAR (dotted yellow) and GFT+VAR (dash-dotted blue), in contrast with CDC's weighted %ILI activity level (solid black).

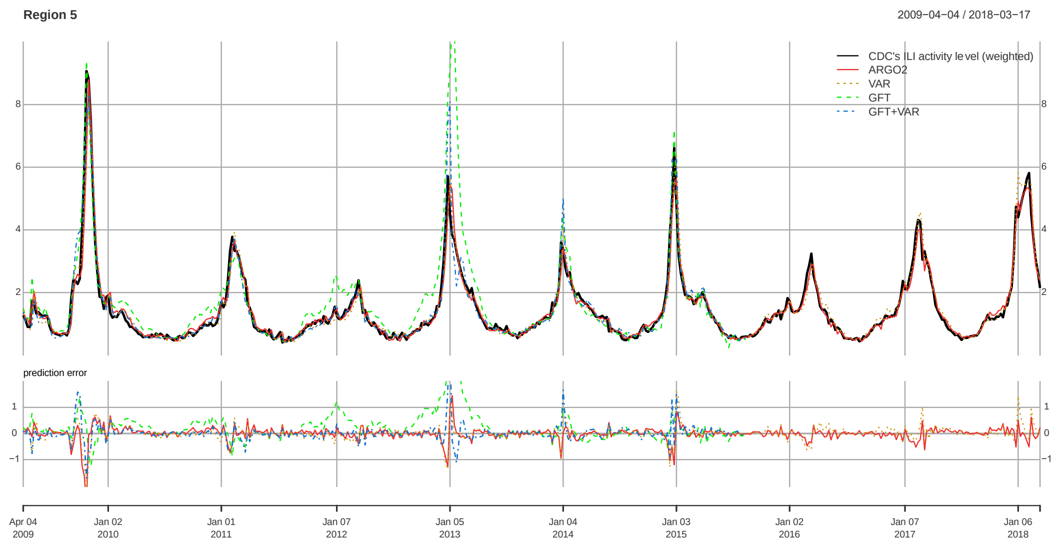

Supplementary Figure S8. Plots of the %ILI estimates (top) and the estimation errors (bottom) for Region 5. The evaluation period is from March 29, 2009 to March 17, 2018. Methods compared include ARGO2 (solid red), GFT (dashed green), VAR (dotted yellow) and GFT+VAR (dash-dotted blue), in contrast with CDC's weighted %ILI activity level (solid black).

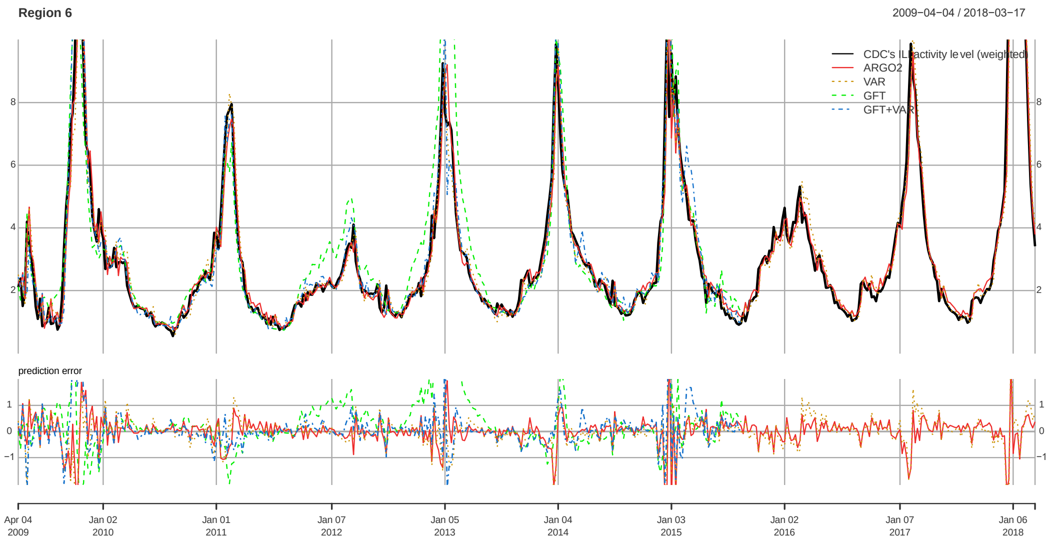

Supplementary Figure S9. Plots of the %ILI estimates (top) and the estimation errors (bottom) for Region 6. The evaluation period is from March 29, 2009 to March 17, 2018. Methods compared include ARGO2 (solid red), GFT (dashed green), VAR (dotted yellow) and GFT+VAR (dash-dotted blue), in contrast with CDC's weighted %ILI activity level (solid black).

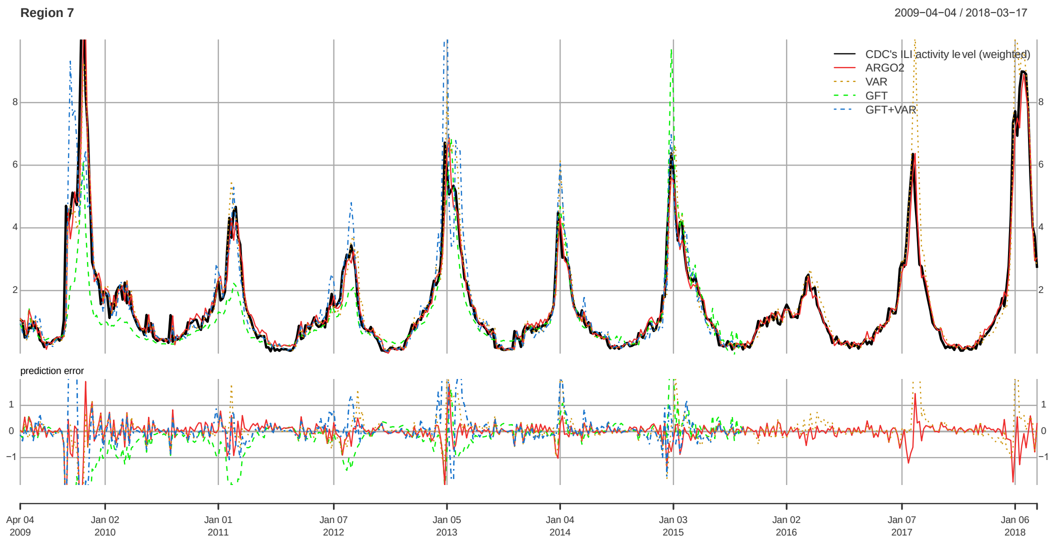

Supplementary Figure S10. Plots of the %ILI estimates (top) and the estimation errors (bottom) for Region 7. The evaluation period is from March 29, 2009 to March 17, 2018. Methods compared include ARGO2 (solid red), GFT (dashed green), VAR (dotted yellow) and GFT+VAR (dash-dotted blue), in contrast with CDC’s weighted %ILI activity level (solid black).

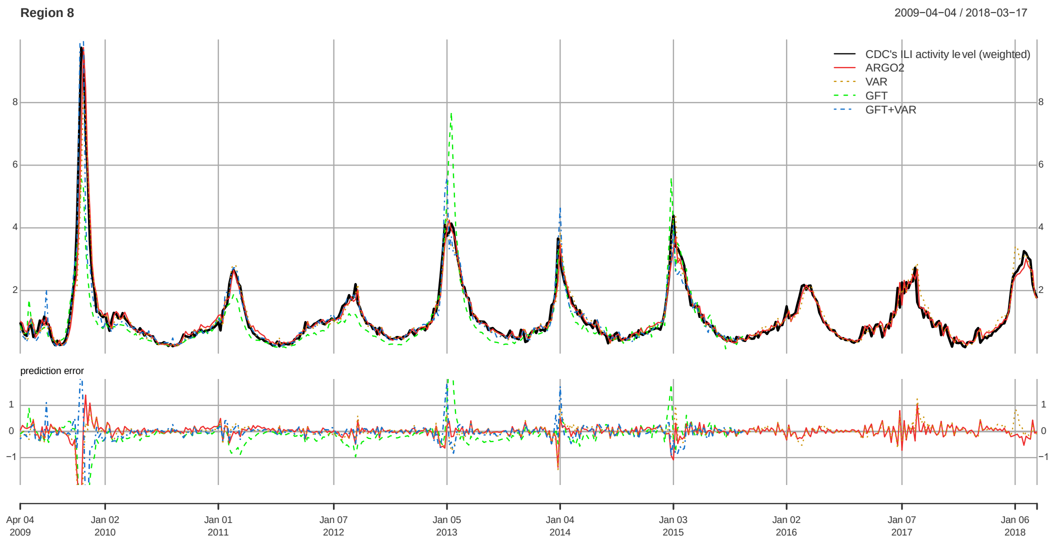

Supplementary Figure S11. Plots of the %ILI estimates (top) and the estimation errors (bottom) for Region 8. The evaluation period is from March 29, 2009 to March 17, 2018. Methods compared include ARGO2 (solid red), GFT (dashed green), VAR (dotted yellow) and GFT+VAR (dash-dotted blue), in contrast with CDC's weighted %ILI activity level (solid black).

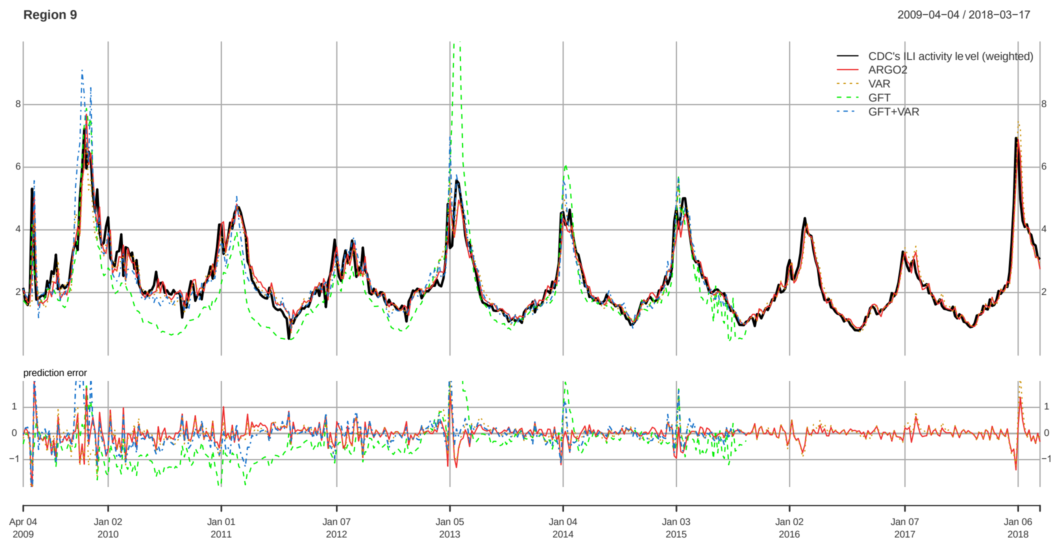

Supplementary Figure S12. Plots of the %ILI estimates (top) and the estimation errors (bottom) for Region 9. The evaluation period is from March 29, 2009 to March 17, 2018. Methods compared include ARGO2 (solid red), GFT (dashed green), VAR (dotted yellow) and GFT+VAR (dash-dotted blue), in contrast with CDC's weighted %ILI activity level (solid black).

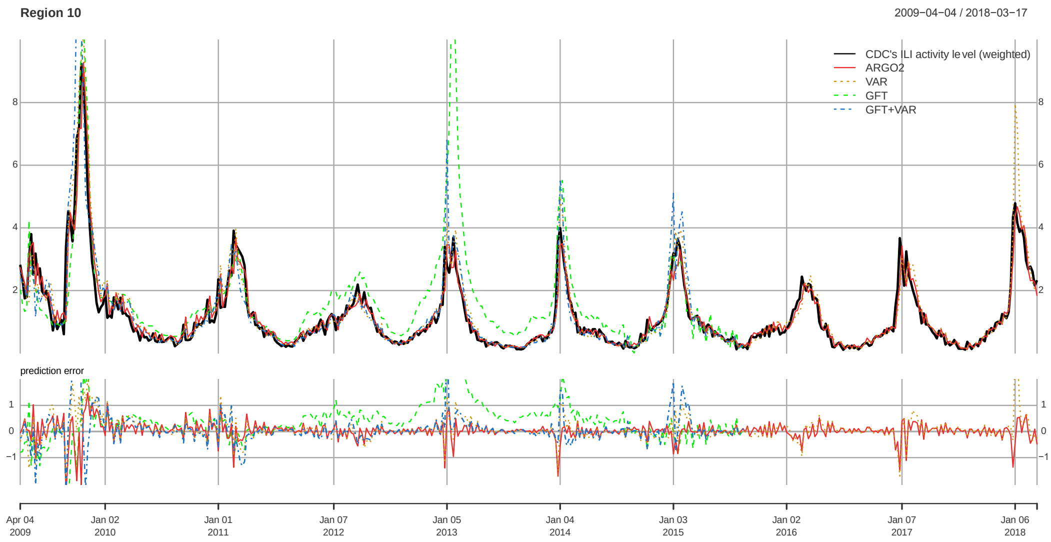

Supplementary Figure S13. Plots of the %ILI estimates (top) and the estimation errors (bottom) for Region 10. The evaluation period is from March 29, 2009 to March 17, 2018. Methods compared include ARGO2 (solid red), GFT (dashed green), VAR (dotted yellow) and GFT+VAR (dash-dotted blue), in contrast with CDC's weighted %ILI activity level (solid black).

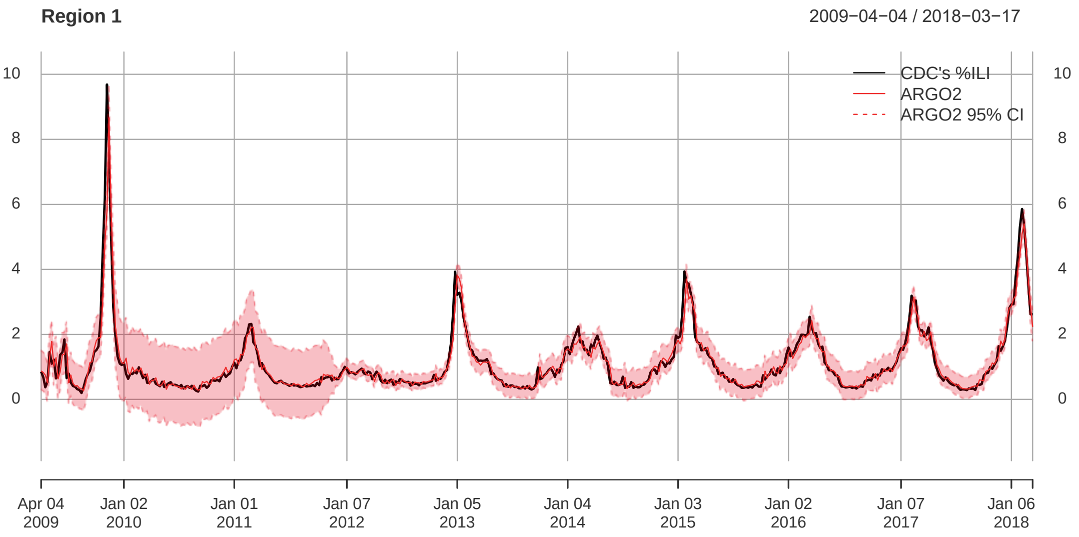

Supplementary Figure S14. Plot of 95% CI coverage by ARGO2 for Region 1. The evaluation period is from March 29, 2009 to March 17, 2018. %ILI estimates by ARGO2 (solid red) with 95% CI constructed by ARGO2 (red shade) are compared with CDC's weighted %ILI activity level (solid black).

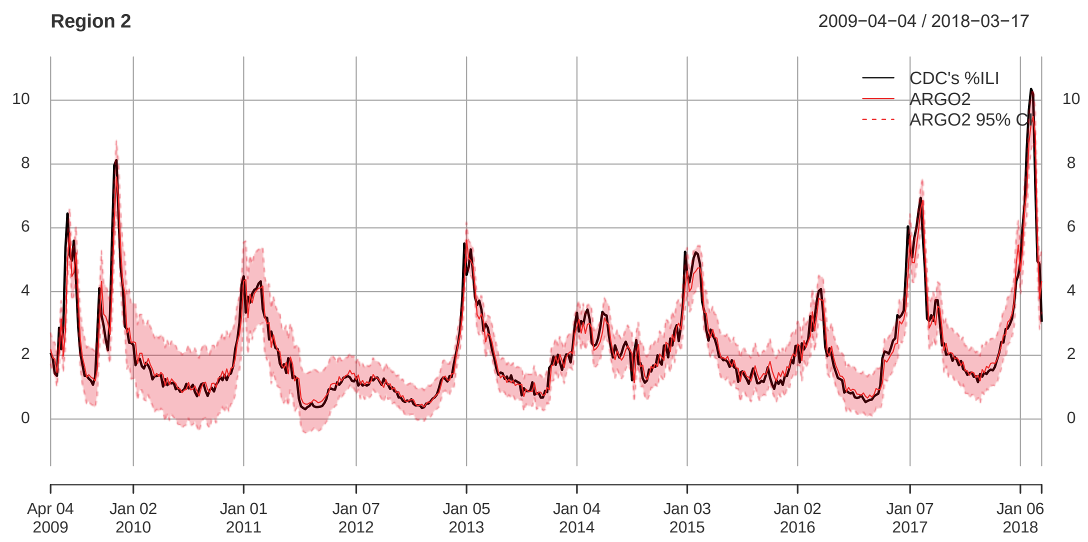

Supplementary Figure S15. Plot of 95% CI coverage by ARGO2 for Region 2. The evaluation period is from March 29, 2009 to March 17, 2018. %ILI estimates by ARGO2 (solid red) with 95% CI constructed by ARGO2 (red shade) are compared with CDC's weighted %ILI activity level (solid black).

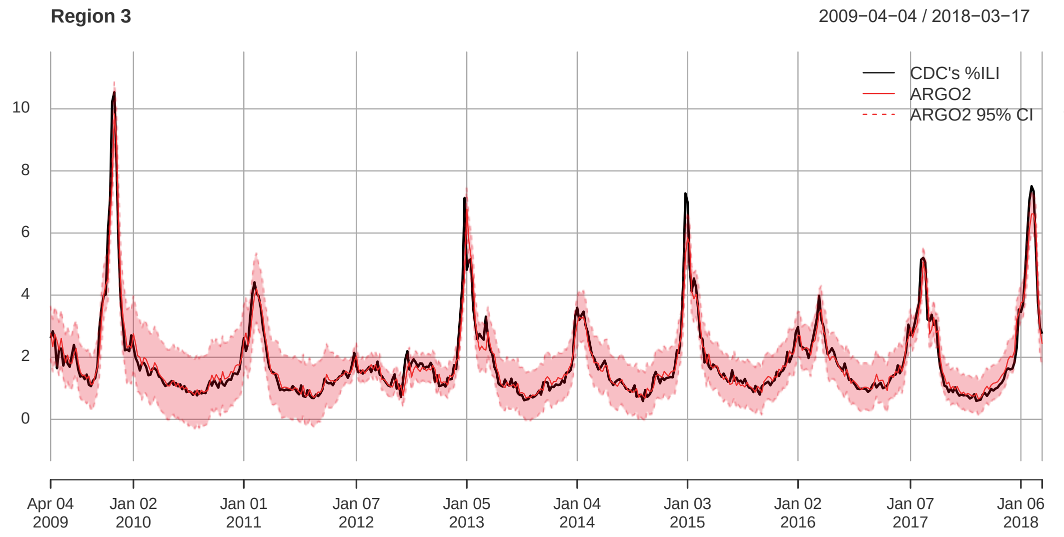

Supplementary Figure S16. Plot of 95% CI coverage by ARGO2 for Region 3. The evaluation period is from March 29, 2009 to March 17, 2018. %ILI estimates by ARGO2 (solid red) with 95% CI constructed by ARGO2 (red shade) are compared with CDC's weighted %ILI activity level (solid black).

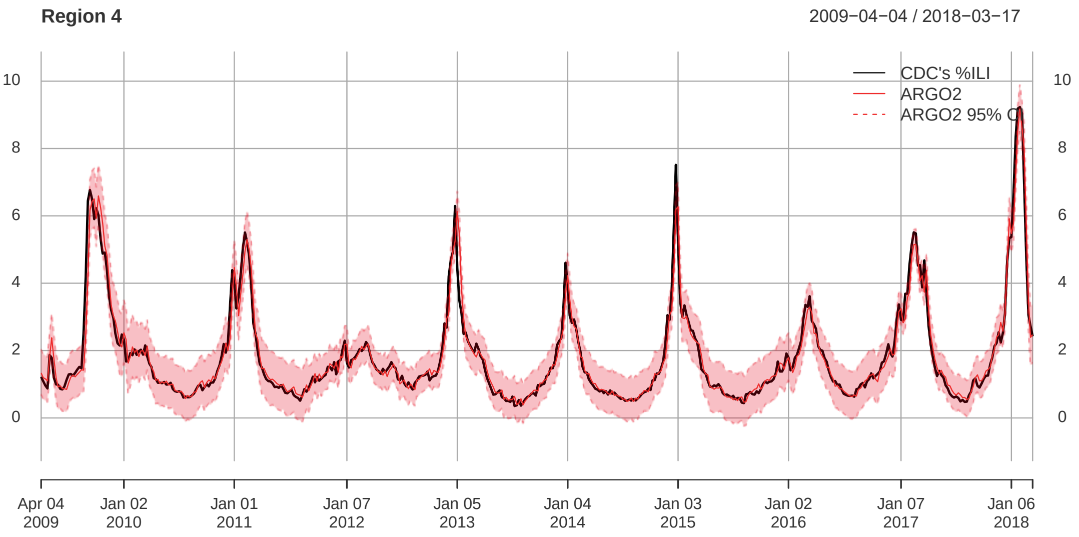

Supplementary Figure S17. Plot of 95% CI coverage by ARGO2 for Region 4. The evaluation period is from March 29, 2009 to March 17, 2018. %ILI estimates by ARGO2 (solid red) with 95% CI constructed by ARGO2 (red shade) are compared with CDC's weighted %ILI activity level (solid black).

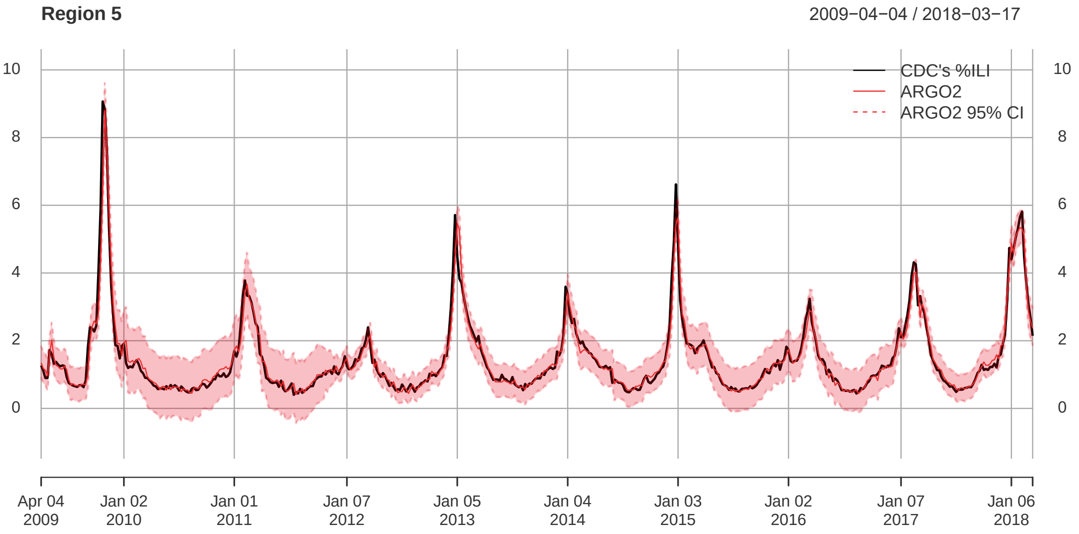

Supplementary Figure S18. Plot of 95% CI coverage by ARGO2 for Region 5. The evaluation period is from March 29, 2009 to March 17, 2018. %ILI estimates by ARGO2 (solid red) with 95% CI constructed by ARGO2 (red shade) are compared with CDC's weighted %ILI activity level (solid black).

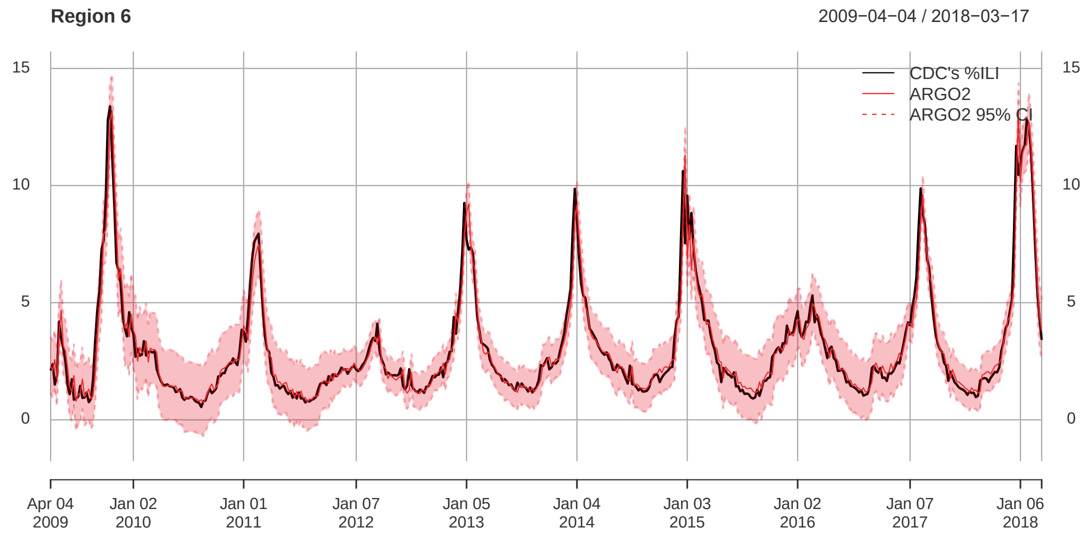

Supplementary Figure S19. Plot of 95% CI coverage by ARGO2 for Region 6. The evaluation period is from March 29, 2009 to March 17, 2018. %ILI estimates by ARGO2 (solid red) with 95% CI constructed by ARGO2 (red shade) are compared with CDC's weighted %ILI activity level (solid black).

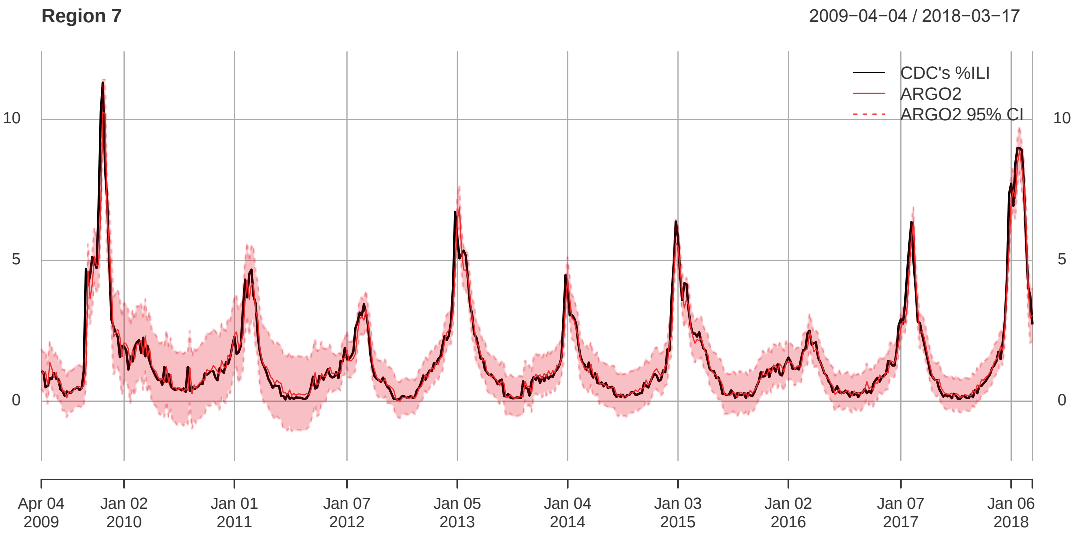

Supplementary Figure S20. Plot of 95% CI coverage by ARGO2 for Region 7. The evaluation period is from March 29, 2009 to March 17, 2018. %ILI estimates by ARGO2 (solid red) with 95% CI constructed by ARGO2 (red shade) are compared with CDC's weighted %ILI activity level (solid black).

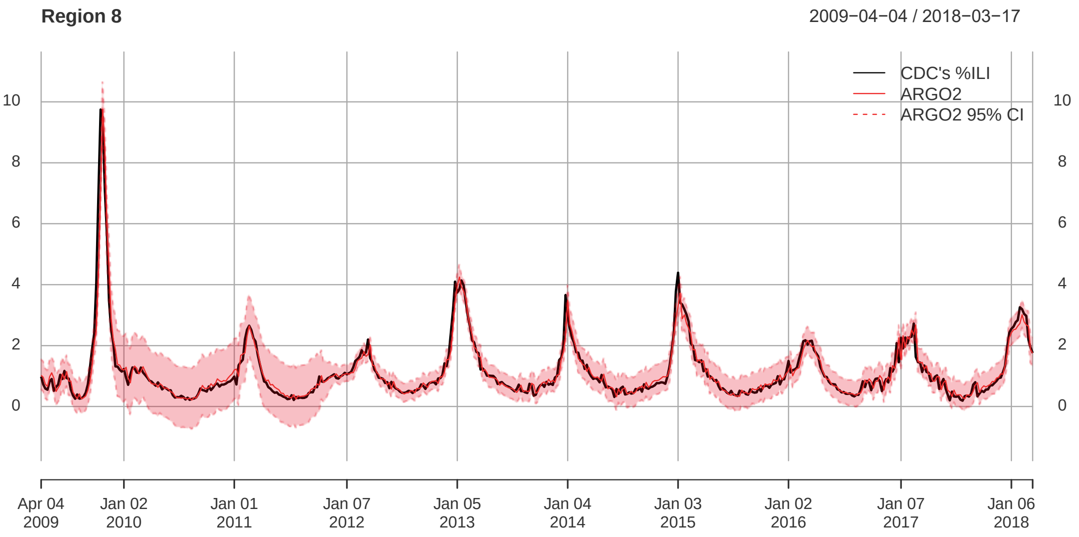

Supplementary Figure S21. Plot of 95% CI coverage by ARGO2 for Region 8. The evaluation period is from March 29, 2009 to March 17, 2018. %ILI estimates by ARGO2 (solid red) with 95% CI constructed by ARGO2 (red shade) are compared with CDC's weighted %ILI activity level (solid black).

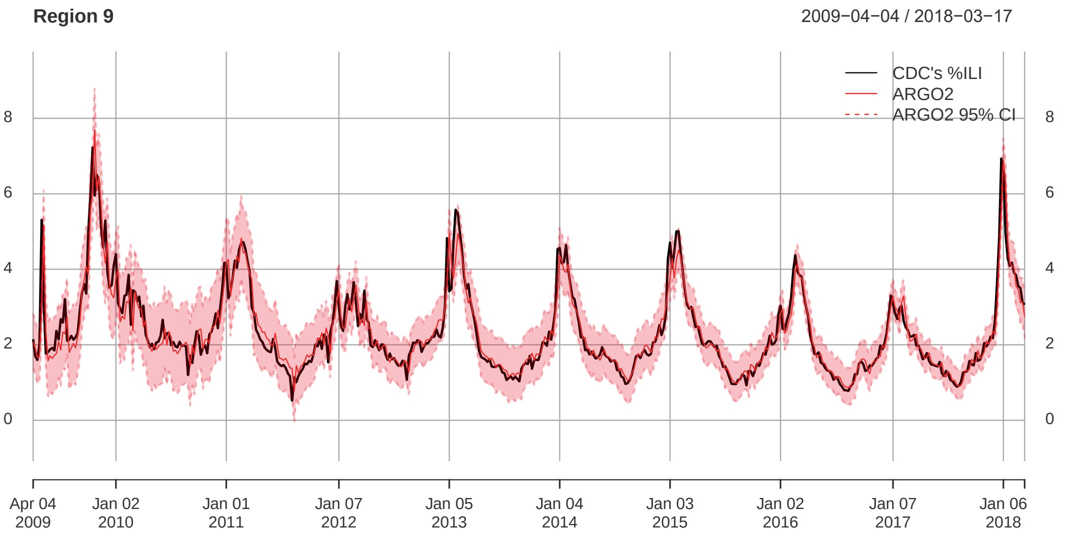

Supplementary Figure S22. Plot of 95% CI coverage by ARGO2 for Region 9. The evaluation period is from March 29, 2009 to March 17, 2018. %ILI estimates by ARGO2 (solid red) with 95% CI constructed by ARGO2 (red shade) are compared with CDC's weighted %ILI activity level (solid black).

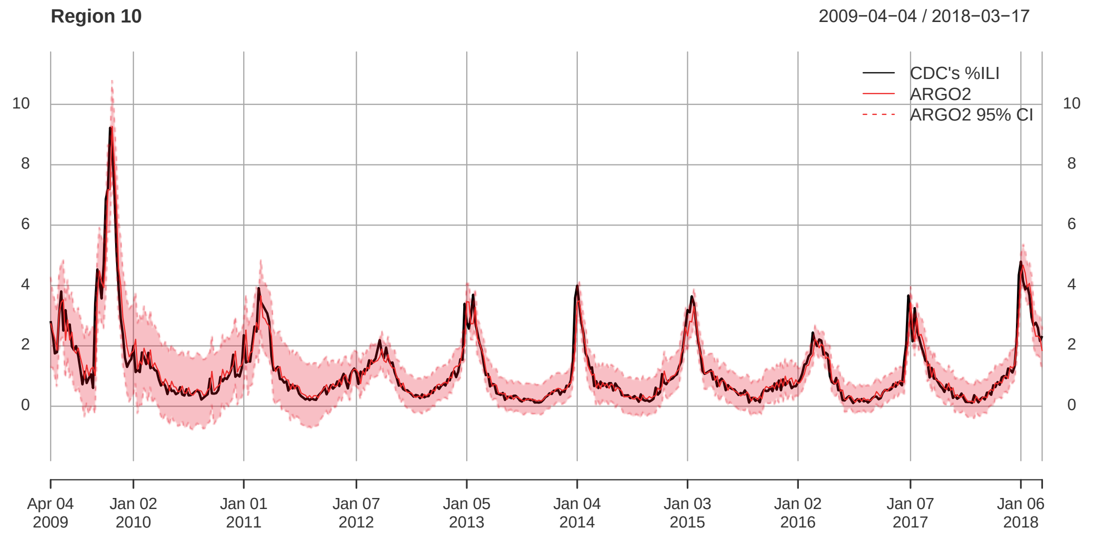

Supplementary Figure S23. Plot of 95% CI coverage by ARGO2 for Region 10. The evaluation period is from March 29, 2009 to March 17, 2018. %ILI estimates by ARGO2 (solid red) with 95% CI constructed by ARGO2 (red shade) are compared with CDC's weighted %ILI activity level (solid black).

Supplementary Table S1. Comparison of different methods for %ILI estimation in US HHS Region 1 (CT, MA, ME, NH, RI, VT).

	Whole period	'09-'15	H1N1	'10-'11	'11-'12	'12-'13	'13-'14	'14-'15	'15-'16	'16-'17	'17-'18
MSE											
ARGO2	**0.658**	**0.684**	0.650	**0.682**	**0.960**	**0.669**	**0.781**	0.820	**0.748**	**0.568**	**0.405**
VAR	0.740	0.758	0.723	1.079	1.092	0.716	1.145	**0.755**	0.969	0.769	0.501
GFT	–	5.369	**0.236**	1.516	5.424	60.762	3.385	1.349	–	–	–
GFT+VAR	–	0.711	0.548	1.006	1.059	1.878	0.852	0.769	–	–	–
naive	1 (0.137)	1 (0.162)	1 (1.041)	1 (0.037)	1 (0.011)	1 (0.131)	1 (0.046)	1 (0.13)	1 (0.045)	1 (0.07)	1 (0.248)
MAE											
ARGO2	**0.839**	**0.850**	0.753	**0.835**	**0.995**	0.798	**0.854**	**0.912**	**0.891**	0.852	**0.589**
VAR	0.877	0.894	0.794	0.987	1.076	**0.793**	1.003	0.923	1.017	**0.813**	0.681
GFT	–	1.738	**0.518**	1.367	2.021	5.932	1.857	1.459	–	–	–
GFT+VAR	–	0.916	0.715	1.001	1.082	1.044	0.956	1.145	–	–	–
naive	1 (0.182)	1 (0.186)	1 (0.615)	1 (0.143)	1 (0.087)	1 (0.224)	1 (0.18)	1 (0.221)	1 (0.166)	1 (0.194)	1 (0.377)
MAPE											
ARGO2	**0.958**	**0.947**	0.851	**0.895**	1.026	0.795	**0.838**	**0.960**	**0.915**	0.863	**0.630**
VAR	0.971	0.990	0.987	0.968	1.061	**0.785**	0.949	1.002	0.976	**0.789**	0.744
GFT	–	1.703	**0.650**	1.463	1.899	4.760	1.713	1.525	–	–	–
GFT+VAR	–	1.042	0.983	0.944	1.047	0.984	0.953	1.395	–	–	–
naive	1 (0.155)	1 (0.166)	1 (0.356)	1 (0.135)	**1** (0.125)	1 (0.137)	1 (0.144)	1 (0.127)	1 (0.134)	1 (0.131)	1 (0.144)
Correlation											
ARGO2	**0.957**	0.940	0.918	0.956	0.692	**0.947**	**0.913**	0.930	**0.939**	**0.965**	**0.983**
VAR	0.951	0.938	0.936	0.959	0.619	0.941	0.862	0.935	0.932	0.955	0.974
GFT	–	0.768	**0.978**	0.911	**0.699**	0.831	0.786	0.917	–	–	–
GFT+VAR	–	**0.944**	0.936	**0.960**	0.695	0.938	0.903	**0.940**	–	–	–
naive	0.934	0.914	0.876	0.932	0.693	0.919	0.884	0.914	0.922	0.936	0.953

The evaluation is conducted in multiple periods and multiple metrics. The ***relative*** MSE, MAE, and MAPE to the naive method (i.e., the ratio of the measures between the evaluated method and the naive method), and the correlation are reported, with the best performance (for each metric in each period) in boldface and the original error metrics for the naive method in parentheses. Methods considered here include ARGO2, VAR, GFT, GFT+VAR, and the naive method. All comparisons are conducted on the original percentage scale of CDC's %ILI. The whole period is March 29, 2009 to March 17, 2018. '2009-2015' is March 29, 2009 to August 15, 2015 following GFT's availability. Columns 4 to 12 correspond to the 2009 off-season H1N1 outbreak, and every regular flu reason (week 40 to week 20 next year, defined by CDC's Morbidity and Mortality

Weekly Report [MMWR] https://www.cdc.gov/nndss/downloads.html, 17’-18’ season up to March 17, 2018) since 2010. Note that the methodology of ARGO2 was frozen on Dec 26, 2016.

Supplementary Table S2. Comparison of different methods for %ILI estimation in US HHS Region 2 (NJ, NY).

	Whole period	'09-'15	H1N1	'10-'11	'11-'12	'12-'13	'13-'14	'14-'15	'15-'16	'16-'17	'17-'18
MSE											
ARGO2	**0.681**	**0.701**	**0.661**	**0.790**	1.395	**0.588**	**0.807**	**0.583**	**0.805**	0.832	0.488
VAR	0.886	1.016	1.047	1.529	1.042	0.729	0.903	0.641	1.012	**0.815**	**0.469**
GFT	–	4.627	1.348	6.721	17.637	22.633	3.278	1.829	–	–	–
GFT+VAR	–	1.128	0.768	1.911	1.952	2.346	1.125	0.901	–	–	–
naive	1 (0.257)	1 (0.23)	1 (1.094)	1 (0.178)	**1** (0.015)	1 (0.248)	1 (0.135)	1 (0.24)	1 (0.146)	1 (0.397)	1 (1.043)
MAE											
ARGO2	**0.905**	**0.886**	**0.824**	**0.888**	1.156	**0.732**	**0.906**	0.822	**0.954**	1.008	0.786
VAR	0.963	0.970	0.953	1.157	**0.972**	0.772	0.921	**0.792**	0.986	1.001	**0.767**
GFT	–	2.131	1.172	3.081	4.541	3.659	1.978	1.350	–	–	–
GFT+VAR	–	1.042	0.833	1.310	1.309	1.058	0.910	1.114	–	–	–
naive	1 (0.309)	1 (0.295)	1 (0.79)	1 (0.304)	1 (0.097)	1 (0.358)	1 (0.309)	1 (0.36)	1 (0.302)	**1** (0.453)	1 (0.675)
MAPE											
ARGO2	1.008	**0.982**	0.853	**0.898**	1.174	**0.730**	**0.884**	0.850	1.049	1.028	0.769
VAR	1.009	0.986	0.914	1.087	**0.984**	0.794	0.934	**0.799**	1.130	1.052	**0.760**
GFT	–	2.272	1.279	2.823	4.596	3.040	2.020	1.326	–	–	–
GFT+VAR	–	1.053	**0.796**	1.138	1.313	0.973	0.916	1.200	–	–	–
naive	**1** (0.137)	1 (0.142)	1 (0.227)	1 (0.119)	1 (0.087)	1 (0.13)	1 (0.133)	1 (0.118)	**1** (0.146)	**1** (0.125)	1 (0.138)
Correlation											
ARGO2	**0.964**	**0.954**	0.893	**0.940**	0.661	**0.960**	0.853	**0.953**	**0.925**	**0.934**	0.967
VAR	0.952	0.932	0.843	0.886	**0.695**	0.953	0.832	0.944	0.914	0.932	**0.968**
GFT	–	0.783	0.877	0.870	0.398	0.915	0.871	0.932	–	–	–
GFT+VAR	–	0.938	**0.924**	0.873	0.611	0.905	**0.872**	0.931	–	–	–
naive	0.948	0.935	0.845	0.927	0.684	0.931	0.831	0.910	0.902	0.914	0.931

The evaluation is conducted in multiple periods and multiple metrics. The ***relative*** MSE, MAE, and MAPE to the naive method (i.e., the ratio of the measures between the evaluated method and the naive method), and the correlation are reported, with the best performance (for each metric in each period) in boldface and the original error metrics for the naive method in parentheses.

Supplementary Table S3. Comparison of different methods for %ILI estimation in US HHS Region 3 (DE, MD, PA, VA, WV).

	Whole period	'09-'15	H1N1	'10-'11	'11-'12	'12-'13	'13-'14	'14-'15	'15-'16	'16-'17	'17-'18
MSE											
ARGO2	**0.531**	**0.537**	0.407	0.623	**0.657**	0.729	**0.454**	0.445	**0.516**	**0.536**	**0.437**
VAR	0.724	0.742	0.826	1.085	0.917	**0.690**	0.706	0.448	0.774	0.820	0.497
GFT	–	2.710	**0.402**	2.218	22.358	7.772	2.707	**0.261**	–	–	–
GFT+VAR	–	0.761	0.569	**0.620**	0.926	1.235	0.755	0.503	–	–	–
naive	1 (0.246)	1 (0.268)	1 (0.951)	1 (0.113)	1 (0.038)	1 (0.616)	1 (0.097)	1 (0.554)	1 (0.103)	1 (0.256)	1 (0.544)
MAE											
ARGO2	**0.816**	**0.800**	**0.643**	0.820	**0.809**	**0.857**	**0.780**	**0.704**	**0.719**	**0.868**	0.822
VAR	0.908	0.928	0.880	1.085	0.834	0.916	0.914	0.745	0.858	0.915	**0.754**
GFT	–	1.976	0.732	1.669	5.780	3.647	2.293	0.725	–	–	–
GFT+VAR	–	0.954	0.867	**0.763**	0.901	1.062	1.021	0.888	–	–	–
naive	1 (0.277)	1 (0.284)	1 (0.629)	1 (0.256)	1 (0.153)	1 (0.482)	1 (0.21)	1 (0.43)	1 (0.259)	1 (0.321)	1 (0.484)
MAPE											
ARGO2	**0.912**	**0.883**	**0.678**	0.983	**0.811**	**0.881**	**0.911**	**0.760**	**0.722**	**0.893**	1.112
VAR	0.968	0.984	0.881	1.122	0.837	0.971	0.962	0.802	0.862	1.013	**0.745**
GFT	–	2.512	0.885	2.328	5.943	4.666	2.925	1.082	–	–	–
GFT+VAR	–	1.025	0.994	**0.841**	0.869	1.009	1.050	1.062	–	–	–
naive	1 (0.132)	1 (0.137)	1 (0.189)	1 (0.122)	1 (0.106)	1 (0.169)	1 (0.108)	1 (0.141)	1 (0.111)	1 (0.138)	1 (0.133)
Correlation											
ARGO2	**0.965**	**0.960**	0.967	**0.972**	**0.785**	0.896	0.973	0.962	**0.933**	**0.958**	**0.982**
VAR	0.951	0.945	0.944	0.955	0.693	**0.899**	0.949	0.950	0.891	0.931	0.969
GFT	–	0.865	**0.972**	0.953	0.732	0.891	**0.975**	**0.985**	–	–	–
GFT+VAR	–	0.947	0.964	0.970	0.738	0.828	0.945	0.944	–	–	–
naive	0.933	0.926	0.913	0.947	0.676	0.859	0.926	0.891	0.871	0.916	0.941

The evaluation is conducted in multiple periods and multiple metrics. The ***relative*** MSE, MAE, and MAPE to the naive method (i.e., the ratio of the measures between the evaluated method and the naive method), and the correlation are reported, with the best performance (for each metric in each period) in boldface and the original error metrics for the naive method in parentheses.

Supplementary Table S4. Comparison of different methods for %ILI estimation in US HHS Region 4 (AL, FL, GA, KY, MS, NC, SC, TN).

	Whole period	'09-'15	H1N1	'10-'11	'11-'12	'12-'13	'13-'14	'14-'15	'15-'16	'16-'17	'17-'18
MSE											
ARGO2	**0.604**	**0.720**	1.082	**0.655**	**0.707**	1.023	**0.414**	0.365	**0.532**	**0.607**	**0.273**
VAR	0.715	0.776	1.097	0.748	1.085	**0.636**	0.924	0.488	1.109	0.906	0.410
GFT	–	3.765	2.676	3.513	1.732	12.655	1.102	**0.238**	–	–	–
GFT+VAR	–	1.259	2.983	0.701	0.866	1.083	0.608	0.551	–	–	–
naive	1 (0.194)	1 (0.177)	**1** (0.345)	1 (0.234)	1 (0.042)	1 (0.323)	1 (0.15)	1 (0.539)	1 (0.075)	1 (0.234)	1 (0.885)
MAE											
ARGO2	**0.801**	**0.839**	1.050	**0.807**	**0.811**	0.933	**0.646**	0.618	**0.773**	**0.811**	**0.527**
VAR	0.930	0.942	1.159	0.816	1.080	**0.818**	0.888	0.757	1.089	0.975	0.698
GFT	–	1.986	1.683	2.029	1.481	4.049	1.307	**0.597**	–	–	–
GFT+VAR	–	1.039	1.602	0.839	0.953	1.036	0.762	0.845	–	–	–
naive	1 (0.252)	1 (0.236)	**1** (0.368)	1 (0.365)	1 (0.157)	1 (0.363)	1 (0.244)	1 (0.428)	1 (0.211)	1 (0.375)	1 (0.651)
MAPE											
ARGO2	**0.881**	**0.889**	1.042	0.857	**0.838**	0.970	**0.698**	**0.664**	**0.773**	**0.819**	**0.519**
VAR	1.001	1.002	1.161	**0.810**	1.044	**0.926**	0.884	0.828	1.106	0.987	0.694
GFT	–	2.183	1.334	2.152	1.453	4.231	1.837	0.834	–	–	–
GFT+VAR	–	1.051	1.226	0.853	0.979	1.128	0.920	1.053	–	–	–
naive	1 (0.123)	1 (0.123)	**1** (0.137)	1 (0.137)	1 (0.096)	1 (0.142)	1 (0.117)	1 (0.145)	1 (0.111)	1 (0.136)	1 (0.159)
Correlation											
ARGO2	**0.973**	**0.962**	0.951	0.963	**0.849**	0.916	0.968	0.959	**0.970**	**0.964**	**0.984**
VAR	0.969	0.961	0.954	0.962	0.789	**0.942**	0.944	0.942	0.918	0.952	0.975
GFT	–	0.837	0.948	**0.975**	0.771	0.892	**0.977**	**0.979**	–	–	–
GFT+VAR	–	0.952	0.953	0.966	0.845	0.910	0.967	0.935	–	–	–
naive	0.956	0.948	**0.955**	0.944	0.793	0.909	0.922	0.881	0.929	0.937	0.940

The evaluation is conducted in multiple periods and multiple metrics. The ***relative*** MSE, MAE, and MAPE to the naive method (i.e., the ratio of the measures between the evaluated method and the naive method), and the correlation are reported, with the best performance (for each metric in each period) in boldface and the original error metrics for the naive method in parentheses.

Supplementary Table S5. Comparison of different methods for %ILI estimation in US HHS Region 5 (IL, IN, MI, MN, OH, WI).

	Whole period	'09-'15	H1N1	'10-'11	'11-'12	'12-'13	'13-'14	'14-'15	'15-'16	'16-'17	'17-'18
MSE											
ARGO2	**0.487**	**0.521**	0.484	**0.631**	**0.617**	0.827	**0.441**	0.327	**0.438**	**0.456**	**0.239**
VAR	0.603	0.590	0.471	0.726	1.039	**0.768**	0.735	0.560	1.113	0.580	0.554
GFT	–	3.483	**0.290**	1.536	6.846	22.519	1.009	**0.178**	–	–	–
GFT+VAR	–	0.751	0.393	0.731	0.745	2.451	1.094	0.448	–	–	–
naive	1 (0.16)	1 (0.182)	1 (0.762)	1 (0.095)	1 (0.041)	1 (0.237)	1 (0.103)	1 (0.397)	1 (0.066)	1 (0.119)	1 (0.314)
MAE											
ARGO2	**0.722**	**0.755**	**0.638**	0.843	**0.811**	**0.826**	**0.701**	0.608	**0.615**	**0.705**	**0.549**
VAR	0.856	0.866	0.769	0.849	1.074	0.837	0.990	0.710	1.021	0.810	0.678
GFT	–	1.754	0.650	1.499	2.842	5.051	1.236	**0.529**	–	–	–
GFT+VAR	–	0.855	0.661	**0.799**	0.837	1.232	1.055	0.665	–	–	–
naive	1 (0.212)	1 (0.215)	1 (0.499)	1 (0.21)	1 (0.157)	1 (0.314)	1 (0.183)	1 (0.367)	1 (0.205)	1 (0.242)	1 (0.395)
MAPE											
ARGO2	**0.825**	**0.865**	**0.717**	0.998	**0.808**	**0.806**	**0.757**	**0.690**	**0.615**	**0.718**	0.710
VAR	0.950	0.959	0.904	0.836	1.032	0.895	1.019	0.727	1.019	0.994	**0.684**
GFT	–	2.000	0.888	2.062	3.174	5.377	1.327	0.777	–	–	–
GFT+VAR	–	0.929	0.849	**0.758**	0.882	1.169	1.130	0.727	–	–	–
naive	1 (0.125)	1 (0.129)	1 (0.18)	1 (0.124)	1 (0.118)	1 (0.127)	1 (0.089)	1 (0.14)	1 (0.125)	1 (0.11)	1 (0.128)
Correlation											
ARGO2	**0.973**	**0.966**	0.963	**0.969**	**0.895**	0.936	0.949	0.961	**0.956**	**0.975**	**0.988**
VAR	0.965	0.961	0.968	0.962	0.857	**0.937**	0.924	0.937	0.876	0.968	0.978
GFT	–	0.880	**0.978**	0.938	0.709	0.906	**0.964**	**0.988**	–	–	–
GFT+VAR	–	0.955	0.968	0.964	0.881	0.906	0.934	0.962	–	–	–
naive	0.944	0.934	0.919	0.947	0.837	0.920	0.883	0.880	0.894	0.938	0.943

The evaluation is conducted in multiple periods and multiple metrics. The ***relative*** MSE, MAE, and MAPE to the naive method (i.e., the ratio of the measures between the evaluated method and the naive method), and the correlation are reported, with the best performance (for each metric in each period) in boldface and the original error metrics for the naive method in parentheses.

Supplementary Table S6. Comparison of different methods for %ILI estimation in US HHS Region 6 (AR, LA, NM, OK, TX).

	Whole period	’09-’15	H1N1	’10-’11	’11-’12	’12-’13	’13-’14	’14-’15	’15-’16	’16-’17	’17-’18
MSE											
ARGO2	**0.677**	**0.734**	**0.660**	0.554	**0.681**	**0.675**	0.578	**0.916**	**0.637**	**0.617**	**0.462**
VAR	0.904	0.914	0.754	0.667	0.801	0.718	0.805	1.225	1.656	0.824	0.770
GFT	–	3.676	0.977	0.922	8.808	21.962	1.061	0.969	–	–	–
GFT+VAR	–	1.112	1.270	**0.399**	1.191	1.321	**0.500**	1.267	–	–	–
naive	1 (0.498)	1 (0.494)	1 (1.385)	1 (0.442)	1 (0.094)	1 (0.592)	1 (0.559)	1 (1.355)	1 (0.203)	1 (0.521)	1 (1.762)
MAE											
ARGO2	**0.822**	**0.852**	0.820	0.842	**0.856**	**0.798**	**0.727**	0.888	**0.799**	**0.746**	**0.587**
VAR	0.923	0.911	**0.820**	0.853	0.971	0.889	0.850	0.892	1.330	0.894	0.765
GFT	–	1.699	0.989	0.953	4.105	4.810	1.092	**0.879**	–	–	–
GFT+VAR	–	0.999	0.993	**0.653**	1.189	1.001	0.787	1.162	–	–	–
naive	1 (0.426)	1 (0.419)	1 (0.89)	1 (0.461)	1 (0.207)	1 (0.534)	1 (0.498)	1 (0.752)	1 (0.362)	1 (0.542)	1 (0.957)
MAPE											
ARGO2	**0.919**	**0.927**	**0.894**	0.902	**0.871**	**0.784**	**0.750**	0.964	**0.811**	**0.823**	**0.581**
VAR	0.989	0.977	0.903	0.879	1.004	0.931	0.852	**0.896**	1.335	0.985	0.719
GFT	–	1.777	1.218	0.837	4.423	4.669	1.219	0.961	–	–	–
GFT+VAR	–	1.036	0.926	**0.674**	1.272	0.925	0.916	1.335	–	–	–
naive	1 (0.132)	1 (0.138)	1 (0.271)	1 (0.134)	1 (0.083)	1 (0.126)	1 (0.105)	1 (0.15)	1 (0.104)	1 (0.13)	1 (0.145)
Correlation											
ARGO2	**0.970**	**0.961**	0.959	0.971	0.899	**0.957**	0.958	0.897	**0.886**	**0.972**	**0.973**
VAR	0.960	0.951	0.955	0.964	0.882	0.954	0.946	0.877	0.726	0.960	0.958
GFT	–	0.879	0.939	0.978	**0.914**	0.943	0.956	**0.928**	–	–	–
GFT+VAR	–	0.955	**0.968**	**0.986**	0.913	0.915	**0.977**	0.889	–	–	–
naive	0.956	0.948	0.940	0.944	0.855	0.933	0.927	0.893	0.831	0.950	0.943

The evaluation is conducted in multiple periods and multiple metrics. The ***relative*** MSE, MAE, and MAPE to the naive method (i.e., the ratio of the measures between the evaluated method and the naive method), and the correlation are reported, with the best performance (for each metric in each period) in boldface and the original error metrics for the naive method in parentheses.

Supplementary Table S7. Comparison of different methods for %ILI estimation in US HHS Region 7 (IA, KS, MO, NE).

	Whole period	'09-'15	H1N1	'10-'11	'11-'12	'12-'13	'13-'14	'14-'15	'15-'16	'16-'17	'17-'18
MSE											
ARGO2	**0.622**	**0.682**	**0.731**	0.634	**0.669**	**0.777**	**0.416**	**0.374**	**0.622**	**0.669**	**0.334**
VAR	1.277	1.023	0.859	**0.594**	1.704	1.591	1.454	0.974	1.393	5.136	0.806
GFT	–	2.600	3.109	4.732	3.091	1.270	0.473	1.892	–	–	–
GFT+VAR	–	2.525	2.509	0.779	1.756	7.049	1.464	0.826	–	–	–
naive	1 (0.309)	1 (0.319)	1 (1.35)	1 (0.245)	1 (0.118)	1 (0.406)	1 (0.219)	1 (0.388)	1 (0.061)	1 (0.304)	1 (1.053)
MAE											
ARGO2	**0.813**	**0.848**	**0.864**	0.853	**0.810**	**0.830**	0.674	**0.643**	**0.783**	**0.773**	**0.570**
VAR	1.018	0.982	0.867	**0.592**	1.222	1.350	1.044	0.954	1.173	1.412	0.860
GFT	–	1.618	2.018	2.233	1.683	1.380	**0.672**	1.196	–	–	–
GFT+VAR	–	1.361	1.635	0.927	1.149	2.544	1.097	0.997	–	–	–
naive	1 (0.309)	1 (0.31)	1 (0.656)	1 (0.354)	1 (0.259)	1 (0.39)	1 (0.298)	1 (0.412)	1 (0.195)	1 (0.393)	1 (0.7)
MAPE											
ARGO2	1.087	1.110	**0.957**	1.041	**0.802**	**0.757**	0.745	**0.732**	**0.799**	**0.815**	**0.591**
VAR	1.105	1.093	0.975	**0.620**	1.193	1.251	0.925	1.005	1.245	1.118	0.953
GFT	–	1.777	1.476	1.955	1.321	1.485	**0.620**	1.216	–	–	–
GFT+VAR	–	1.194	1.361	0.928	1.040	2.103	1.086	1.122	–	–	–
naive	**1** (0.275)	**1** (0.281)	1 (0.273)	1 (0.19)	1 (0.186)	1 (0.143)	1 (0.199)	1 (0.177)	1 (0.154)	1 (0.205)	1 (0.181)
Correlation											
ARGO2	**0.969**	**0.958**	0.941	0.951	0.946	0.945	0.958	**0.975**	**0.913**	**0.957**	**0.985**
VAR	0.944	0.939	0.938	**0.961**	0.874	**0.948**	0.921	0.944	0.860	0.906	0.975
GFT	–	0.847	**0.955**	0.960	**0.953**	0.934	**0.964**	0.963	–	–	–
GFT+VAR	–	0.867	0.782	0.935	0.943	0.871	0.953	0.944	–	–	–
naive	0.950	0.939	0.920	0.917	0.919	0.929	0.898	0.919	0.858	0.937	0.946

The evaluation is conducted in multiple periods and multiple metrics. The ***relative*** MSE, MAE, and MAPE to the naive method (i.e., the ratio of the measures between the evaluated method and the naive method), and the correlation are reported, with the best performance (for each metric in each period) in boldface and the original error metrics for the naive method in parentheses.

Supplementary Table S8. Comparison of different methods for %ILI estimation in US HHS Region 8 (CO, MT, ND, SD, UT, WY).

	Whole period	’09-’15	H1N1	’10-’11	’11-’12	’12-’13	’13-’14	’14-’15	’15-’16	’16-’17	’17-’18
MSE											
ARGO2	**0.660**	**0.638**	**0.634**	0.719	0.806	**0.483**	0.645	0.628	**0.667**	**0.908**	**0.573**
VAR	0.961	0.950	1.065	**0.587**	1.038	0.763	0.950	**0.381**	1.199	0.966	1.177
GFT	–	2.390	2.151	2.792	4.784	7.130	**0.571**	1.375	–	–	–
GFT+VAR	–	0.924	0.830	0.630	**0.507**	1.826	1.028	0.876	–	–	–
naive	1 (0.132)	1 (0.16)	1 (0.901)	1 (0.052)	1 (0.031)	1 (0.117)	1 (0.14)	1 (0.15)	1 (0.035)	1 (0.136)	1 (0.07)
MAE											
ARGO2	**0.851**	**0.829**	**0.762**	0.978	0.879	**0.693**	**0.799**	0.868	**0.830**	0.951	**0.862**
VAR	0.921	0.890	0.835	0.822	1.075	0.863	1.074	**0.661**	1.084	**0.930**	1.160
GFT	–	1.586	1.371	1.688	2.745	2.100	1.035	1.207	–	–	–
GFT+VAR	–	0.978	0.870	**0.803**	**0.772**	1.153	1.053	1.060	–	–	–
naive	1 (0.19)	1 (0.197)	1 (0.551)	1 (0.162)	1 (0.124)	1 (0.241)	1 (0.226)	1 (0.239)	1 (0.147)	1 (0.273)	1 (0.18)
MAPE											
ARGO2	**0.961**	0.952	0.859	1.267	0.860	**0.745**	**0.870**	0.998	**0.828**	0.975	**0.973**
VAR	0.978	**0.949**	**0.815**	0.858	1.102	0.816	1.143	**0.728**	1.122	**0.932**	1.386
GFT	–	1.545	1.148	1.453	2.811	1.876	1.183	1.364	–	–	–
GFT+VAR	–	0.999	0.852	**0.803**	**0.812**	1.050	1.151	1.063	–	–	–
naive	1 (0.169)	1 (0.162)	1 (0.287)	1 (0.144)	1 (0.099)	1 (0.122)	1 (0.15)	1 (0.134)	1 (0.125)	1 (0.222)	1 (0.105)
Correlation											
ARGO2	**0.961**	**0.963**	0.952	0.969	0.897	**0.979**	0.910	0.967	**0.957**	0.829	**0.987**
VAR	0.943	0.946	0.933	**0.973**	0.895	0.972	0.877	**0.974**	0.920	**0.851**	0.965
GFT	–	0.860	**0.957**	0.929	0.872	0.926	**0.955**	0.930	–	–	–
GFT+VAR	–	0.949	0.946	0.965	**0.937**	0.932	0.905	0.954	–	–	–
naive	0.941	0.941	0.925	0.941	0.877	0.955	0.867	0.931	0.935	0.803	0.965

The evaluation is conducted in multiple periods and multiple metrics. The ***relative*** MSE, MAE, and MAPE to the naive method (i.e., the ratio of the measures between the evaluated method and the naive method), and the correlation are reported, with the best performance (for each metric in each period) in boldface and the original error metrics for the naive method in parentheses.

Supplementary Table S9. Comparison of different methods for %ILI estimation in US HHS Region 9 (AZ, CA, HI, NV).

	Whole period	'09-'15	H1N1	'10-'11	'11-'12	'12-'13	'13-'14	'14-'15	'15-'16	'16-'17	'17-'18
MSE											
ARGO2	**0.747**	**0.778**	0.723	0.891	**0.760**	**0.872**	**0.595**	**0.741**	**0.649**	**0.927**	**0.527**
VAR	0.950	0.934	0.965	**0.652**	0.770	1.237	0.662	0.759	1.080	1.507	0.943
GFT	–	5.283	**0.638**	9.328	1.678	21.771	2.370	1.995	–	–	–
GFT+VAR	–	1.533	1.739	1.591	0.878	1.755	1.332	1.200	–	–	–
naive	1 (0.194)	1 (0.223)	1 (0.833)	1 (0.146)	1 (0.192)	1 (0.296)	1 (0.148)	1 (0.146)	1 (0.097)	1 (0.044)	1 (0.46)
MAE											
ARGO2	**0.895**	**0.911**	**0.882**	0.951	0.847	**0.911**	**0.775**	0.856	**0.742**	**0.944**	**0.775**
VAR	1.007	1.001	1.025	**0.815**	**0.792**	1.254	0.826	**0.848**	0.987	1.106	0.989
GFT	–	2.318	0.910	3.653	1.358	3.664	1.554	1.574	–	–	–
GFT+VAR	–	1.203	1.289	1.229	0.850	1.156	1.240	1.098	–	–	–
naive	1 (0.271)	1 (0.296)	1 (0.613)	1 (0.298)	1 (0.368)	1 (0.354)	1 (0.253)	1 (0.26)	1 (0.246)	1 (0.165)	1 (0.425)
MAPE											
ARGO2	**0.944**	**0.957**	**0.896**	1.056	0.854	**0.929**	**0.821**	**0.868**	**0.738**	**0.971**	**0.811**
VAR	1.049	1.053	1.015	**0.851**	**0.798**	1.492	0.845	0.934	0.970	1.107	0.984
GFT	–	2.361	0.937	3.654	1.359	3.000	1.424	1.826	–	–	–
GFT+VAR	–	1.193	1.223	1.090	0.879	1.349	1.175	1.174	–	–	–
naive	1 (0.108)	1 (0.116)	1 (0.175)	1 (0.103)	1 (0.145)	1 (0.107)	1 (0.093)	1 (0.083)	1 (0.099)	1 (0.076)	1 (0.112)
Correlation											
ARGO2	**0.942**	**0.929**	0.886	0.937	0.766	**0.906**	**0.963**	**0.959**	**0.956**	**0.941**	**0.945**
VAR	0.927	0.915	0.839	**0.953**	0.762	0.881	0.953	0.957	0.924	0.930	0.919
GFT	–	0.809	**0.937**	0.905	**0.844**	0.900	0.937	0.917	–	–	–
GFT+VAR	–	0.888	0.841	0.918	0.755	0.865	0.915	0.932	–	–	–
naive	0.925	0.912	0.831	0.924	0.728	0.894	0.926	0.931	0.933	0.932	0.897

The evaluation is conducted in multiple periods and multiple metrics. The ***relative*** MSE, MAE, and MAPE to the naive method (i.e., the ratio of the measures between the evaluated method and the naive method), and the correlation are reported, with the best performance (for each metric in each period) in boldface and the original error metrics for the naive method in parentheses.

Supplementary Table S10. Comparison of different methods for %ILI estimation in US HHS Region 10 (AK, ID, OR, WA).

	Whole period	'09-'15	H1N1	'10-'11	'11-'12	'12-'13	'13-'14	'14-'15	'15-'16	'16-'17	'17-'18
MSE											
ARGO2	**0.736**	**0.747**	**0.717**	0.786	**0.861**	**0.784**	**0.573**	**0.705**	**0.871**	**0.766**	**0.533**
VAR	1.204	1.040	0.982	0.984	0.959	1.179	0.856	1.407	1.243	0.988	2.903
GFT	–	5.614	1.156	**0.734**	7.047	40.757	3.458	1.092	–	–	–
GFT+VAR	–	2.398	2.833	1.282	0.938	3.044	0.920	4.316	–	–	–
naive	1 (0.185)	1 (0.206)	1 (0.951)	1 (0.243)	1 (0.056)	1 (0.203)	1 (0.219)	1 (0.105)	1 (0.074)	1 (0.227)	1 (0.314)
MAE											
ARGO2	**0.876**	**0.870**	**0.826**	**0.925**	**0.831**	**0.892**	**0.714**	**0.727**	**0.977**	**0.876**	**0.824**
VAR	1.095	1.082	1.036	1.118	0.945	1.211	0.963	1.178	1.168	0.940	1.382
GFT	–	2.380	1.071	1.061	2.872	7.600	2.722	1.024	–	–	–
GFT+VAR	–	1.249	1.373	1.237	0.897	1.273	0.976	1.774	–	–	–
naive	1 (0.255)	1 (0.268)	1 (0.744)	1 (0.335)	1 (0.208)	1 (0.286)	1 (0.28)	1 (0.248)	1 (0.208)	1 (0.314)	1 (0.348)
MAPE											
ARGO2	**0.986**	**0.997**	**0.932**	**0.989**	**0.816**	**0.909**	**0.787**	**0.784**	1.008	0.901	**0.824**
VAR	1.063	1.066	1.189	1.060	0.911	1.285	0.930	1.109	1.124	**0.898**	1.064
GFT	–	3.329	0.998	1.286	3.108	7.836	3.712	1.044	–	–	–
GFT+VAR	–	1.073	1.228	1.121	0.843	1.217	0.937	1.415	–	–	–
naive	1 (0.249)	1 (0.241)	1 (0.274)	1 (0.21)	1 (0.192)	1 (0.228)	1 (0.235)	1 (0.191)	**1** (0.217)	1 (0.218)	1 (0.161)
Correlation											
ARGO2	**0.952**	**0.951**	0.925	0.883	0.860	0.910	0.936	**0.957**	**0.902**	**0.880**	**0.954**
VAR	0.943	0.950	**0.933**	0.858	0.863	0.911	0.930	0.948	0.860	0.847	0.930
GFT	–	0.806	0.919	**0.919**	**0.887**	**0.911**	**0.974**	0.933	–	–	–
GFT+VAR	–	0.909	0.861	0.804	0.857	0.880	0.944	0.916	–	–	–
naive	0.936	0.936	0.898	0.859	0.850	0.887	0.883	0.933	0.892	0.854	0.916

The evaluation is conducted in multiple periods and multiple metrics. The ***relative*** MSE, MAE, and MAPE to the naive method (i.e., the ratio of the measures between the evaluated method and the naive method), and the correlation are reported, with the best performance (for each metric in each period) in boldface and the original error metrics for the naive method in parentheses.

Supplementary Table S11. All search terms identified by Google Correlate as of March 28, 2009.

acute.bronchitis	body.temperature	break.a.fever	bronchitis
cold.or.flu	cold.vs.flu	cough.fever	cure.the.flu
dangerous.fever	fever.cough	fever.flu	fever.reducer
flu.contagious.period	flu.contagious	flu.duration	flu.fever
flu.how.long	flu.in.children	flu.incubation	flu.medicine
flu.or.cold	flu.report	flu.test	flu.treatment
flu.treatments	flu.vs.cold	get.over.the.flu	high.fever
how.long.is.the.flu.contagious	how.long.is.the.flu	how.to.treat.the.flu	incubation.period.for.the.flu
influenza.a.and.b	influenza.a	influenza.contagious	influenza.incubation.period
influenza.incubation	influenza.symptoms	influenza.treatment	influenza.type.a
is.flu.contagious	low.body	normal.body.temperature	normal.body
over.the.counter.flu	painful.cough	pneumonia	reduce.a.fever
remedies.for.the.flu	robitussin	signs.of.the.flu	sinus.infections
sinus	strep	symptoms.of.bronchitis	symptoms.of.flu
symptoms.of.influenza	symptoms.of.pneumonia	symptoms.of.the.flu	treat.flu
treat.the.flu	treating.flu	treating.the.flu	treatment.for.flu
treatment.for.the.flu	tussin	tussionex	type.a.influenza
upper.respiratory	walking.pneumonia		

Supplementary Table S12. Additional terms identified by Google Correlate as of May 22, 2010.

a.influenza	braun.thermoscan	chest.cold	cold.and.flu
cold.versus.flu	contagious.flu	cure.flu	do.i.have.the.flu
ear.thermometer	early.flu.symptoms	expectorant	exposed.to.flu
fight.the.flu	flu.and.cold	flu.and.fever	flu.care
flu.children	flu.complications	flu.cough	flu.germs
flu.headache	flu.incubation.period	flu.lasts	flu.length
flu.recovery	flu.relief	flu.remedies	flu.remedy
flu.reports	flu.symptoms	flu.versus.cold	get.rid.of.the.flu
having.the.flu	how.long.contagious	how.long.does.flu.last	how.long.does.the.flu.last
how.long.flu	how.long.is.flu.contagious	how.to.get.rid.of.the.flu	how.to.treat.flu
human.temperature	i.have.the.flu	incubation.period.for.flu	medicine.for.flu
medicine.for.the.flu	oscillococcinum	over.the.counter.flu.medicine	rapid.flu
reduce.fever	remedies.for.flu	respiratory.flu	signs.of.flu
strep.throat	taking.temperature	tessalon	the.flu.virus
the.flu	thermoscan	what.to.do.if.you.have.the.flu	

Supplementary Table S13. Comparison of different methods in CDC's 2015-2016 Epidemic Prediction Initiative (FluSight challenge) for weekly regional-level flu nowcast.

	Region 1	Region 2	Region 3	Region 4	Region 5	Region 6	Region 7	Region 8	Region 9	Region 10	Overall
MSE											
4Sight	3.950	0.721	4.771	13.878	7.562	3.796	14.572	7.833	1.020	3.666	3.120
ARETE	0.857	**0.315**	1.961	5.695	3.093	1.701	5.622	3.170	0.601	2.138	1.336
CU1	2.860	0.958	3.255	3.056	2.867	2.682	3.407	2.144	0.862	3.006	1.721
CU2	1.034	1.328	2.058	1.650	1.456	1.303	1.973	1.267	0.689	2.110	1.309
Delphi-Archefilter	1.892	0.759	1.281	2.013	1.309	2.961	1.887	1.028	**0.310**	1.487	1.186
Delphi-Epicast	0.933	0.722	0.810	1.147	0.802	1.573	1.205	**0.584**	0.464	**0.612**	0.828
Delphi-Stat	1.195	0.734	0.919	0.886	0.754	2.346	1.369	0.672	0.384	1.529	0.975
ISU	3.050	1.584	6.484	4.715	7.560	3.952	5.904	3.963	2.083	2.297	2.865
JL	1.340	0.874	1.910	4.066	5.326	1.399	3.010	3.369	0.936	2.910	1.527
KBSI1	1.835	1.230	1.328	1.045	1.431	1.885	1.335	0.752	0.661	1.208	1.220
NEU	1.908	1.355	2.720	2.687	1.586	3.244	1.665	1.268	0.588	0.910	1.605
PSI	3.965	0.746	2.130	2.210	2.575	1.858	2.809	1.186	1.152	0.971	1.367
UMN	3.815	0.333	2.544	4.630	6.307	3.594	6.203	4.728	1.667	3.135	2.072
ARGO2	**0.814**	0.761	**0.634**	**0.453**	**0.429**	**0.765**	**0.725**	0.673	0.731	0.906	**0.731**
VAR	0.852	0.890	0.654	1.494	1.511	1.809	1.949	1.253	0.661	0.815	1.042
naive	1 (0.085)	1 (1.081)	1 (0.146)	1 (0.124)	1 (0.073)	1 (0.360)	1 (0.102)	1 (0.055)	1 (0.530)	1 (0.132)	1 (0.269)

We report the ***relative*** MSE of the participants' estimation to the naive method, i.e., numbers showing are the ratio of the MSE of each method over that of the naive method, for nowcasting at each region, as well as the average over the ten US HHS regions. A value higher than 1 indicates worse estimation accuracy than the naïve method. We also include ARGO2, VAR and naive method for comparison. All results are calculated based on *unrevised* CDC data. The original MSE for the naive method is in the parentheses.

Supplementary Table S14. Point estimate and 95% Confidence Interval (CI) of Relative Efficiency based on average MSE of ten US HHS regions, comparing the benchmark methods to ARGO2.

	Point Estimate	95% CI
GFT	5.66	[1.16, 22.70]
VAR+GFT	1.99	[1.05, 3.14]
VAR	1.32	[1.13, 1.54]
naive	1.47	[1.13, 1.89]

Relative Efficiency being larger than one indicates higher estimation accuracy of ARGO2 compared to the benchmark method.

Supplementary Table S15. Comparison of different methods for regional %ILI estimation in additional metrics.

	Whole period	’09-’15	H1N1	’10-’11	’11-’12	’12-’13	’13-’14	’14-’15	’15-’16	’16-’17	’17-’18
MSE+											
ARGO2	**0.487**	**0.535**	**0.436**	**0.571**	**0.595**	**0.926**	**0.404**	**0.459**	**0.526**	**0.505**	**0.217**
VAR	0.965	0.856	0.483	0.882	1.018	1.091	1.486	1.318	1.128	1.544	0.769
GFT	–	7.395	0.536	–	4.228	30.078	3.968	1.671	–	–	–
GFT+VAR	–	2.171	2.138	0.723	1.294	4.453	2.309	1.601	–	–	–
naive	1 (0.201)	1 (0.198)	1 (0.755)	1 (0.175)	1 (0.068)	1 (0.22)	1 (0.119)	1 (0.301)	1 (0.1)	1 (0.246)	1 (0.941)
MSE-											
ARGO2	**0.821**	0.831	0.838	0.916	0.879	0.669	0.608	0.745	**0.778**	0.870	0.634
VAR	0.868	0.889	0.966	**0.746**	1.104	0.734	0.576	0.515	1.245	**0.755**	**0.531**
GFT	–	1.545	1.293	3.213	–	–	–	**0.345**	–	–	–
GFT+VAR	–	**0.761**	**0.768**	0.947	**0.705**	**0.666**	**0.292**	0.507	–	–	–
naive	1 (0.263)	1 (0.293)	1 (1.294)	1 (0.19)	1 (0.061)	1 (0.455)	1 (0.261)	1 (0.541)	1 (0.083)	1 (0.235)	1 (0.576)
Bias											
ARGO2	**-0.001**	**0.000**	-0.055	0.042	-0.008	-0.011	-0.042	-0.047	-0.020	-0.042	**-0.041**
VAR	0.009	-0.011	-0.151	0.031	-0.040	0.053	-0.015	-0.005	0.019	0.109	0.085
GFT	–	0.057	-0.354	-0.362	0.196	1.299	0.057	0.005	–	–	–
GFT+VAR	–	0.029	0.113	-0.103	0.035	0.182	0.008	0.051	–	–	–
naive	-0.001	0.004	**-0.009**	**0.001**	**-0.005**	**0.005**	**-0.003**	**-0.001**	**-0.006**	**-0.005**	-0.068

The evaluation is based on the average of ten US HHS regions in multiple periods and multiple metrics. The ***relative*** MSE+ and MAE- to the naive method (i.e., the ratio of the error metric of a specific method over that of the naive method) and the bias are reported, with the best performance, for each metric in each period, in boldface and the original error metrics for the naive method in parentheses. Methods considered here include ARGO2, VAR, GFT, GFT+VAR and the naive method. All comparisons are conducted on the original scale of CDC’s %ILI. The whole period is March 29, 2009 to March 17, 2018. “2009-2015” is March 29, 2009 to August 15, 2015 following GFT’s availability. Columns 4 to 12 correspond to the 2009 off-season H1N1 outbreak, and every post-2009 regular flu reason (week 40 to week 20 next year, defined by CDC’s Morbidity and Mortality Weekly Report, 17’-18’ season up to March 17, 2018). Note that the methodology of ARGO2 was frozen on Dec 26, 2016.

Supplementary Table S16. Comparison of different methods for regional %ILI estimation in relative measures.

	Whole period	'09-'15	H1N1	'10-'11	'11-'12	'12-'13	'13-'14	'14-'15	'15-'16	'16-'17	'17-'18
MSE											
ARGO2	**0.644**	**0.680**	**0.654**	**0.677**	**0.742**	**0.757**	**0.559**	**0.620**	**0.653**	**0.679**	**0.415**
VAR	0.918	0.889	0.866	0.831	1.036	0.900	0.905	0.834	1.201	1.421	0.755
GFT	–	3.851	1.286	3.206	5.623	17.945	1.660	0.903	–	–	–
GFT+VAR	–	1.353	1.385	0.910	1.095	2.371	0.887	0.966	–	–	–
naive	1 (0.231)	1 (0.242)	1 (0.961)	1 (0.179)	1 (0.064)	1 (0.317)	1 (0.182)	1 (0.4)	1 (0.09)	1 (0.231)	1 (0.669)
MAE											
ARGO2	**0.836**	**0.847**	**0.802**	**0.870**	**0.857**	**0.831**	**0.755**	**0.762**	**0.809**	**0.848**	**0.663**
VAR	0.954	0.951	0.907	0.900	0.986	0.974	0.937	0.842	1.079	0.989	0.829
GFT	–	1.925	1.096	1.926	2.698	4.100	1.531	0.989	–	–	–
GFT+VAR	–	1.072	1.072	0.952	0.979	1.259	0.966	1.050	–	–	–
naive	1 (0.268)	1 (0.271)	1 (0.636)	1 (0.289)	1 (0.182)	1 (0.355)	1 (0.268)	1 (0.372)	1 (0.23)	1 (0.327)	1 (0.519)
MAPE											
ARGO2	**0.965**	**0.969**	**0.869**	0.992	**0.872**	**0.837**	**0.804**	**0.820**	**0.846**	**0.882**	**0.736**
VAR	1.019	1.016	0.969	**0.903**	0.998	1.026	0.948	0.893	1.095	0.981	0.863
GFT	–	2.169	1.069	1.908	2.803	4.371	1.892	1.156	–	–	–
GFT+VAR	–	1.070	1.037	0.921	0.973	1.193	1.016	1.161	–	–	–
naive	1 (0.161)	1 (0.163)	1 (0.237)	1 (0.142)	1 (0.124)	1 (0.143)	1 (0.137)	1 (0.14)	1 (0.133)	1 (0.149)	1 (0.141)

The evaluation is based on the average of ten US HHS regions in multiple periods and multiple metrics. The ***relative*** MSE, MAE, and MAPE to the naive method (i.e., the ratio of the error metric of a specific method over that of the naive method) are reported, with the best performance, for each metric in each period, in boldface and the original error metrics for the naive method in parentheses. Methods considered here include ARGO2, VAR, GFT, GFT+VAR and the naive method. All comparisons are conducted on the original scale of CDC's %ILI. The whole period is March 29, 2009 to March 17, 2018. "2009-2015" is March 29, 2009 to August 15, 2015 following GFT's availability. Columns 4 to 12 correspond to the 2009 off-season H1N1 outbreak, and every post-2009 regular flu reason (week 40 to week 20 next year, defined by CDC's Morbidity and Mortality Weekly Report, 17'-18' season up to March 17, 2018). Note that 2017-2018 is the validation period as the methodology of ARGO2 was frozen on December 26, 2016.
